# Association between the no-reflow phenomenon and clinical outcomes after endovascular treatment for acute ischemic stroke: A systematic review and meta-analysis

**DOI:** 10.1093/esj/23969873251376846

**Published:** 2026-01-01

**Authors:** Anderson Matheus Pereira da Silva, Ocílio Ribeiro Gonçalves, Luciano Falcão, Filipe Virgilio Ribeiro, Mariana Lee Han, Isabelle Rodrigues Menezes, Elizabeth Honorato de Farias, Julie Loiola, Gabriel Marinheiro, Gustavo Sousa Noleto, Johannes Kaesmacher, Adnan Mujanovic, Ahmet Günkan

**Affiliations:** Department of Pharmacy, Federal University of Vale do São Francisco, Petrolina, PE, Brazil; Department of Medicine, Federal University of Piauí, Teresina, PI, Brazil; Department of Medicine, Bahiana School of Medicine and Public Health, Salvador, BA, Brazil; Department of Medicine, Faculty of Medicine, Barão de Mauá University Center, Ribeirão Preto, SP, Brazil; Department of Medicine, University of São Paulo, São Paulo, São Paulo, SP, Brazil; Department of Medicine, State University of Rio Grande do Norte, Mossoró, RN, Brazil; Department of Medicine, Federal University of Roraima, Boa Vista, RR, Brazil; Neurology Department, University of Louisville, Louisville, KY, USA; Department of Medicine, Federal University of Ceará, Sobral, CE, Brazil; Department of Neurosurgery, University of São Paulo, São Paulo, SP, Brazil; Department of Diagnostic and Interventional Neuroradiology, University Hospital Bern, Inselspital, University of Bern, Bern, Switzerland; Department of Diagnostic and Interventional Neuroradiology, University Hospital Bern, Inselspital, University of Bern, Bern, Switzerland; Division of Vascular and Interventional Radiology, Department of Medical Imaging, University of Arizona, Tucson, AZ, USA

**Keywords:** No-reflow phenomenon, endovascular treatment, acute ischemic stroke, large vessel occlusion, intracranial hemorrhage, meta-analysis

## Abstract

**Background:**

The no-reflow phenomenon, characterized by impaired microvascular reperfusion despite successful macrovascular recanalization, has been identified as a potential contributor to poor outcomes in acute ischemic stroke (AIS) treated with endovascular therapy (EVT). This systematic review and meta-analysis aimed to assess the prevalence and clinical impact of no-reflow phenomenon in AIS patients undergoing EVT.

**Methods:**

We conducted a systematic review and meta-analysis of randomized controlled trials (RCTs) and observational studies reporting the no-reflow phenomenon after EVT. Databases searched included PubMed, Embase, and CENTRAL (inception to February 9, 2025). Outcomes included no-reflow prevalence, functional outcomes (mRS), early neurological recovery, infarct volume, hemorrhagic complications, and 90-day mortality. Pooled risk ratios (RR) or mean differences (MD) were calculated using random-effects meta-analysis, and heterogeneity was assessed with *I*^2^.

**Results:**

Eight studies (*n* = 1483 patients) were included. The pooled prevalence of no-reflow was 20.5% (95% CI 6.2%–49.9%; *I*^2^ = 96.9%). Compared with controls, patients with no-reflow had reduced early neurological recovery (RR 0.76; 95% CI 0.64–0.90) and increased risk of hemorrhagic transformation (RR 1.82; 95% CI 1.18–2.79) and symptomatic intracranial hemorrhage (RR 1.88; 95% CI 1.00–3.56). Differences in functional independence (mRS 0–2) and mortality were not statistically significant. Subgroup analyses based on study design revealed divergent patterns, particularly for infarct volume, which was significantly greater in no-reflow patients in post-hoc RCTs but not in the overall analysis.

**Conclusion:**

No-reflow affects one in five EVT-treated patients and is associated with adverse neurological and hemorrhagic outcomes. Findings highlight the need for standardized definitions and prospective trials to clarify its clinical impact.

## Graphical Abstract

**Graphical Abstract:**
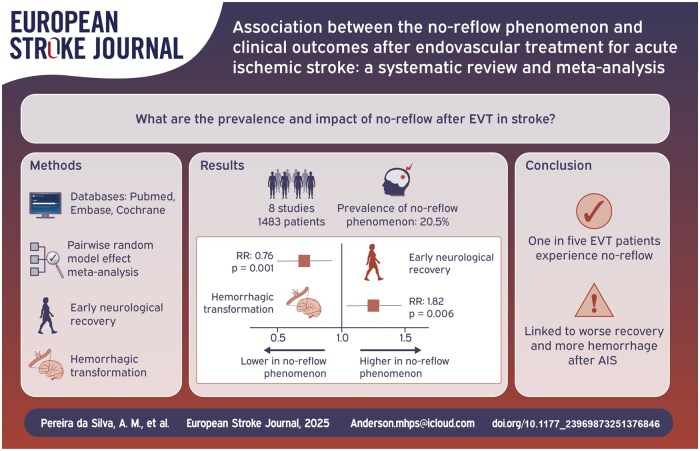

## Introduction

Acute ischemic stroke (AIS) due to large vessel occlusion (LVO) is a major global health burden, accounting for significant morbidity and mortality. With the advent of endovascular treatment (EVT), stroke care has advanced remarkably, with current international guidelines strongly recommending EVT for eligible patients with anterior circulation LVO within 24 h of symptom onset,^1–3^ and for select posterior circulation strokes; these recommendations are supported by reperfusion rates of up to 90% in clinical practice.^1^ However, despite successful macrovascular reperfusion, up to half of EVT-treated patients experience poor functional recovery, a condition referred to as futile recanalization (FR).^4^

One of the mechanisms behind FR is the no-reflow phenomenon, characterized by impaired microvascular perfusion despite successful macrovascular reperfusion.^5,6^ This phenomenon affects 25%–35% of EVT-treated patients and is independently associated with infarct progression, hemorrhagic transformation, and poor clinical outcomes.^7^ Pathophysiologically, it is hypothesized that no-reflow phenomenon results from microvascular obstruction due to endothelial swelling, pericyte contraction, neutrophil plugging, or distal thrombi, leading to persistent tissue hypoxia and secondary brain injury.^8,9^

Although EVT is the current therapy standard for LVO-related AIS, its inability to address microcirculatory dysfunction highlights a therapeutic and neuropathologic gap.^10^ Even with prompt and technically successful EVT, patients with no-reflow phenomenon are at increased risk of early neurological worsening and intracerebral hemorrhage.^5–7,11^ Angiographic markers (modified Capillary Index Score) and perfusion imaging (flat-panel, CT or MR perfusion) can identify no-reflow phenomenon, but these tools are not routinely available nor standardized across clinical settings.^7,11^

Current literature shows heterogeneity in how no-reflow phenomenon is defined (angiographic scores vs perfusion thresholds like rCBV < 34% or Tmax > 6 s), limiting data comparability.^11,12^ Moreover, while early associations with hemorrhagic transformation and neurological deterioration are established, the prognostic value of no-reflow phenomenon for 90-day disability or mortality remains unclear there are also no established therapies aimed at reversing or preventing no-reflow phenomenon, though preclinical evidence suggests roles for anti-inflammatory or vasodilatory agents.^5^

This systematic review and meta-analysis aims to clarify the prevalence and clinical impact of the no-reflow phenomenon in AIS patients treated with EVT.

## Methods

This systematic review and meta-analysis was reported according to the Cochrane Handbook and the Preferred Reporting Items for Systematic Review and Meta-Analysis (PRISMA) 2020 statement.^13,14^ The protocol was registered in the International Prospective Register of Systematic Reviews (PROSPERO), registration number CRD420251015325.

### Eligibility criteria

The included studies met the following criteria: (1) randomized controlled trials (RCTs) or cohort studies, either prospective or retrospective in design; (2) enrolled individuals diagnosed with AIS undergoing EVT for LVO; (3) presented a group with probable no-reflow phenomenon, defined as blockage of local blood vessels resulting in ischemia of the tissue in the downstream target territory, despite subsequent reopening of the vessels^15^; (4) had a minimum 90-day follow-up; and (4) reported at least one outcome of interest. The studies were excluded if published only as editorial letters, conference abstracts, or studies with overlapping populations. We excluded studies that did not report clinical outcomes specific to patients with no-reflow. Studies were also excluded if they provided an inadequate or ambiguous definition of the no-reflow phenomenon or failed to meet the predefined PICOT criteria.

### Search strategy, study selection, and quality assessment

We systematically searched the following databases up to 09, February 2025: PubMed, Embase, and Cochrane Central Register of Controlled Trials (CENTRAL). The search strategy was composed of the following keywords and boolean operators: (“No-reflow”) AND (Stroke) AND (Thrombectomy OR Endovascular). Forward and backward reference searching complemented the database searches. The risk of bias for RCTs was assessed using Version 2 of the Cochrane risk-of-bias tool (RoB 2), while Risk of Bias in Nonrandomized Studies of Interventions tool (ROBINS-I) was applied to observational studies.^16,17^ Two authors independently assessed the studies for data extraction and quality assessment (A.S. & O.R.), and any conflict was resolved by a third author (A.G.) and consensus agreement. Publication bias was evaluated through visual inspection of funnel plots and assessed quantitatively using Egger’s regression test.

### Outcomes, definitions and subanalysis

The outcomes of interest were prevalence of no-reflow, functional outcomes at 90 days (modified Rankin Scale (mRS)), reperfusion rates, early neurological recovery, baseline lesion volume (mL), NIHSS at 24 h, delta NIHSS in 24 h, hemorrhagic transformation, symptomatic intracranial hemorrhage ICH (sICH)^18^ and mortality at 90 days.^19^ The prevalence of no-reflow was defined as the proportion of patients who developed the no-reflow phenomenon following endovascular treatment, calculated as the number of no-reflow cases divided by the total number of patients who underwent EVT in each study. Functional independence was defined as mRS 0–2, and excellent outcome as mRS 0–1, according to established criteria.^20^ Successful macrovascular reperfusion was graded using the expanded Treatment in Cerebral Infarction (eTICI) score and defined as eTICI 2b–3 or mTICI 2c–3, according to the classification applied in each study. sICH was defined according to the criteria used in each included study. The specific definitions adopted are presented in Table S1.

We performed subgroup analyses for each outcome based on study design (observational studies vs RCTs), aiming to explore potential methodological sources of heterogeneity across outcomes.

### Statistical analysis

A single-proportion analysis was conducted to estimate the prevalence of the no-reflow phenomenon. risk ratios (RR) were calculated for binary outcomes and mean differences (MD) for continuous outcomes, both with 95% confidence intervals (CI), using a random-effects model with the Mantel–Haenszel method. *p*-Values < 0.05 were considered statistically significant. Heterogeneity was assessed using the *Q* test and its *p*-value, with the magnitude evaluated by *I*^2^ values: 0%–40% indicating low, 40%–60% moderate, and >60% high heterogeneity.^13^ Statistical analysis was performed using R 4.4.2 (R Foundation for Statistical Computing, Vienna, Austria) with the meta package, employing the inverse variance and the DerSimonian and Laird methods.^21^

## Results

### Study selection

**Figure 1.**
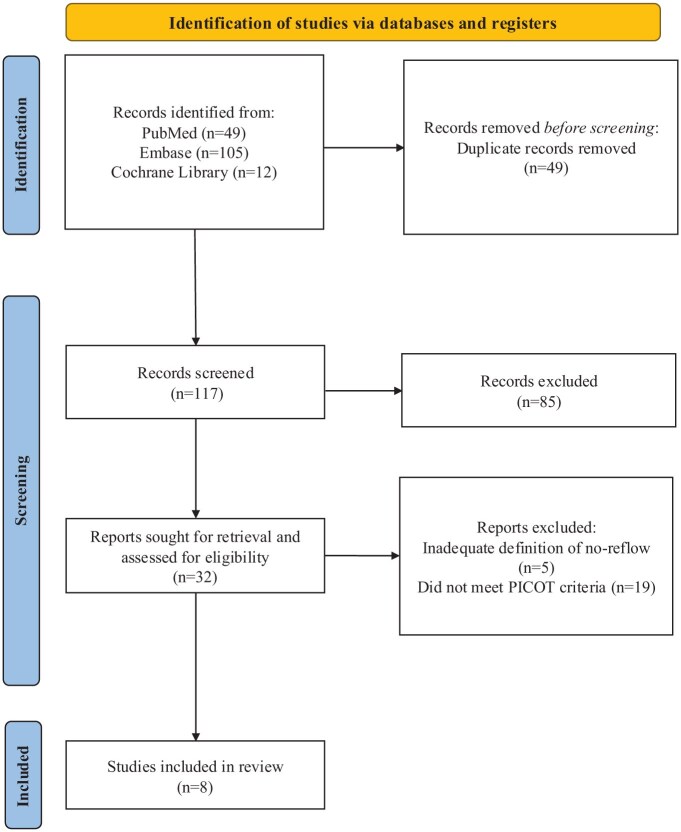
PRISMA flow diagram

A total of 166 records were identified through database searches, including PubMed (*n* = 49), Embase (*n* = 105), and the Cochrane Library (*n* = 12). After removing 49 duplicates, 117 records remained for title and abstract screening. Of these, 85 were excluded based on predefined eligibility criteria. Full-text assessment was conducted for 32 articles. Subsequently, 24 were excluded: five due to inadequate definitions of the no-reflow phenomenon, and 19 for not meeting the prespecified PICOT framework. Eight studies^1,6,7,20,22–25^ were included in the final analysis. The selection process is detailed in Figure 1.

### Baseline characteristics of the included studies and patients

The final analysis incorporated eight studies conducted across seven countries: Australia, New Zealand, Japan, Italy, Germany, China, and France. A total of 1483 patients were included, with 424 assigned to the no-reflow group and 1059 to the group without no-reflow. The majority of the studies were observational, either retrospective (*n* = 4) or prospective (*n* = 2) cohorts, with only two RCTs. Follow-up durations, where specified, ranged from 3 to 90 days. The median age across the no-reflow groups varied between 69.6 and 85 years, while in the group without no-reflow, it ranged from 62.9 to 73 years. Comorbidities such as hypertension were present in up to 77.5% of patients, and diabetes mellitus in up to 33.3%. Atrial fibrillation was reported in 36.7% to 57.8% of patients depending on the cohort. Baseline stroke severity, measured by the NIHSS, showed median scores ranging from 12 to 19, and initial infarct size (basal core volume) varied widely, with median values between 8.5 and 81 mL. The ASPECTS score, reported in seven cohorts, ranged from 7 to 9. Procedural times such as onset to puncture and puncture to recanalization ranged from 103 to 472 min and 30 to 70.1 min, respectively, where available. Prior administration of tPA was documented in 19.2% to 64% of patients. Complete baseline characteristics are detailed in Table 1.

**Table 1.** Baseline characteristics of included studies.

Author, year	Group	Intervention (*n*)	Study design	Country	Follow-up, months	Age, Median (IQR)	HTN, *n*/total (%)	DM, *n*/total (%)	DLP, *n*/total (%)	AF, *n*/total (%)	History of TIA or Stroke, *n*/total (%)	NIHSS, Median (IQR)	ASPECTS, Median (IQR)	Basal core (mL), Median (IQR)	Onset to Puncture (min), Median (IQR)	Puncture to Recanalization (min), Median (IQR)	Prior tPA, *n*/total (%)
Ng, 2021	No-Reflow	33	Post-hoc analysis of RCT	Australia, New Zealand	0.8	74 (66.5–83.5)	18/33 (54.5%)	7/33 (21.2%)	NA	13/33 (39.4%)	NA	17 (15–21)	NA	9.0 (3–34)	255 (200–290)	NA	NA
	No No-Reflow	97				73 (65–82)	65/97 (67.0%)	13/97 (13.4%)	NA	41/97 (42.3%)	NA	16 (12–21)	NA	8.5 (0–21.9)	207 (152–269)	NA	NA
Horie, 2025	No-Reflow	52	PC	Japan	3	NA	NA	NA	NA	NA	NA	NA	NA	NA	NA	NA	NA
Nicolini, 2023	No-Reflow	65	RC	Italy	3	69.6 ± 12.9,*	46/65 (70.8%)	7/65 (10.8%)	19/65 (29.2%)	31/65 (48.4%)	8/65 (12.3%)	15.2 ± 6.9,*	8.0 ± 1.4,*	17.0 ± 21.2,*	456.0 ± 245.2,*	69.9 ± 42.6,*	28/65 (43.1%)
	No No-Reflow	120				72.7 ± 13.7,*	93/120 (77.5%)	26/120 (21.7%)	40/120 (33.3%)	58/120 (48.3%)	14/120 (11.7%)	14.1 ± 5.8,*	8.2 ± 1.5,*	19.1 ± 38.3,*	468.5 ± 278.2,*	70.1 ± 38.7,*	54/120 (45.4%)
Petzsche, 2024	No-Reflow	10	PC	Germany	3	85 (71–91)	9/10 (90%)	1/10 (10%)	4/10 (40%)	4/10 (40%)	2/10 (20%)	14 (6–16)	9 (7–10)	81 (15–224)	472 (263–1155)	NA	4/10 (40%)
	No No-Reflow	101				72 (61–81)	69/101 (69%)	20/101 (20%)	21/101 (21%)	55/101 (55%)	21/101 (21%)	12 (7–17)	9 (7–10)	51 (12–133)	275 (180–543)	NA	43/101 (43%)
Ng, 2018	No-Reflow	53	RO	Australia	3	75 (66–83)	38/53 (71.7%)	9/53 (17.0%)	29/53 (54.7%)	29/53 (54.7%)	6/53 (11.3%)	15 (9–19)	NA	46.0 ± 67.0,*	193 (154–301)	NA	31/53 (58.5%)
	No No-Reflow	53				NA	31/53 (58.5%)	7/53 (13.2%)	25/53 (47.2%)	6/53 (11.3%)	9/53 (17.0%)	NA	NA	31.8 ± 44.0,*	NA	NA	31/53 (58.5%)
Zhao, 2022	No-Reflow (High PI)	45	RC	China	3	69.69 ± 12.89,*	29/45 (64.6%)	12/45 (26.7%)	10/45 (22.2%)	26/45 (57.8%)	6/45 (13.3%)	14.5 (11–19)	9 (8–10)	NA	358 (301–442)	NA	13/45 (28.9%)
	No No-Reflow (Lower PI)	125				62.91 ± 12.17,*	78/125 (62.4%)	26/125 (20.8%)	27/125 (21.6%)	61/125 (48.8%)	30/125 (24.0%)	13.0 (10–17)	9 (8–10)	NA	409 (322–518)	NA	44/125 (35.2%)
Schiphorst, 2021	No-Reflow	1	PC	France	3	NA	NA	NA	NA	1/1 (100%)	NA	1/1 (100%)	NA	NA	160	NA	1/1 (100%)
	No No-Reflow	32				70 (61–79)	18/33 (55%)	NA	NA	19/33 (58%)	NA	18 (12–21)	NA	10 (4–26)	150 (124–202)	30 (16–46)	21/33 (64%)
Rivet, 2025	No-Reflow	30	Post-hoc analysis of RCT	Australia & New Zealand	3	73 (66–84)	15/30 (50.0%)	7/30 (23.3%)	NA	11/30 (36.7%)	NA	17 (15–21)	NA	9 (3–33)	103 (70–144)	NA	NA
	No No-Reflow	426				NA	273/426 (64.0%)	66/426 (15.5%)	NA	131/426 (30.7%)	NA	NA	NA	NA	NA	NA	NA

^*^Mean ± SD.

AF: Atrial Fibrillation; DM: Diabetes Mellitus; DLP: Dyslipidemia; HTN: Hypertension; PC: Prospective Cohort; RC: Retrospective Cohort; RO: Retrospective Observational; RCT: Randomized Controlled Trial; N: number of patients; NA: Not Available.

### Diagnostic criteria for no-reflow phenomenon

Among the seven studies included in this meta-analysis, the definition and diagnostic criteria for the no-reflow phenomenon varied substantially with respect to imaging modality and timing of evaluation. Perfusion imaging at 24 h post-intervention was used in three studies,^6,20,25^ while DSA immediately following thrombectomy was employed in Nicolini 2023 to identify microvascular flow deficits. Two studies^23,24^ utilized TCD within 24 to 72 h post-procedure to detect residual hypoperfusion despite successful macrovascular recanalization (TICI ⩾ 2b). Hernandez Petzsche et al.^22^ defined no-reflow based on follow-up MRI findings acquired within 10 days of mechanical thrombectomy. In all cases, patient selection was restricted to those with anterior circulation LVO and angiographic reperfusion rated as mTICI 2b, 2c, or 3. Despite this consistent anatomical and procedural context, the heterogeneity in both diagnostic modality (DSA, TCD, MRI, perfusion CT) and timing of evaluation introduces variability in outcome ascertainment. This methodological diversity limits the comparability across studies and should be considered a potential source of bias in the pooled estimates. Full diagnostic definitions and criteria for each study are detailed in Table S1.

### Pooled analysis of all studies

#### Prevalence of no-reflow

**Figure 2.**
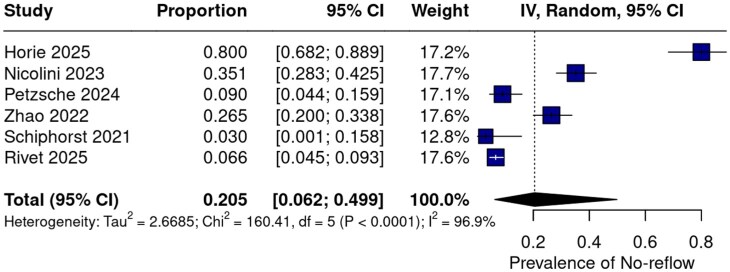
Prevalence of the no-reflow phenomenon across included studies. Blue squares indicate the weight of each study, with larger squares reflecting greater statistical weight. The black diamond represents the pooled prevalence estimate. CI: confidence interval; IV: inverse variance.

Across six studies^1,7,20,22,24,25^ the pooled prevalence of the no-reflow phenomenon was 20.5% (95% CI 6.2%–49.9%; *Q* = 160.41; *p* < 0.0001; *I*^2^ = 96.9%, Figure 2), indicating substantial between-study heterogeneity. Two studies, Ng et al.^23^ and Ng et al.^6^ were excluded due to overlapping populations with Rivet et al.^25^

#### Functional outcomes

**Figure 3.**
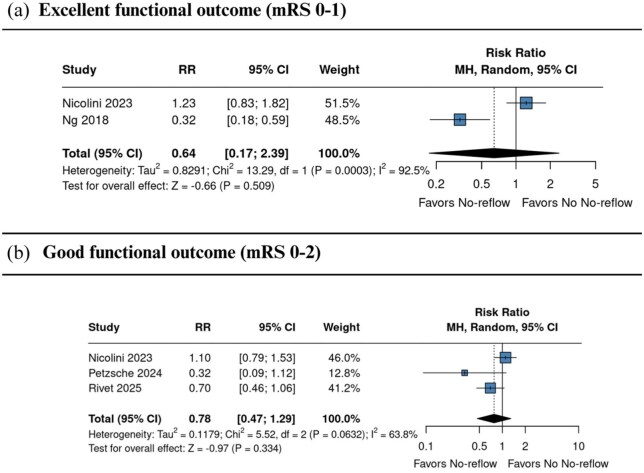
Comparison between no-reflow and no no-reflow, in terms of excellent (a) and good (b) functional outcomes in patients with and without the no-reflow phenomenon. Effect estimates are expressed as RRs with 95% confidence intervals, weighted by study precision. CI: confidence interval; RR: risk ratio.

Two studies^1,23^ reported excellent outcome (mRS 0–1) at 90 days, with no statistically significant difference between groups (RR 0.64, 95% CI 0.17–2.39; *I*^2^ = 92.5%; Figure 3(a)). Three studies^1,22,25^ assessed functional independence (mRS 0–2) at 90 days, also showing no significant difference (RR 0.78, 95% CI 0.47–1.29; *I*^2^ = 63.8%; Figure 3(b)).

#### Functional recovery and death at 90 days

**Figure 4.**
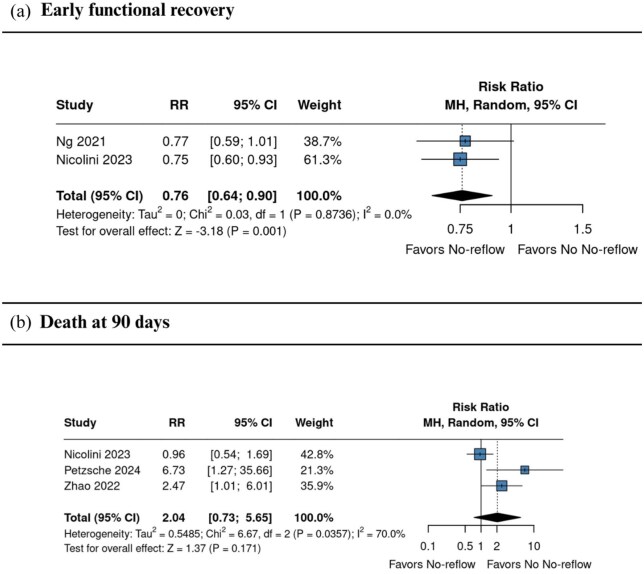
Comparison between no reflow and no no-reflow, in terms of early neurological recovery (a), and mortality at 90 days (b) in patients with and without the no-reflow phenomenon. Effect estimates are expressed as RRs with 95% confidence intervals, weighted by study precision. CI: confidence interval; RR: risk ratio.

Two studies^1,6^ reported early neurological recovery, which occurred less frequently in the no-reflow group (RR 0.76, 95% CI 0.64–0.90; *I*^2^ = 0.0%; Figure 4(a)). Three studies^1,22,24^ evaluated all-cause mortality at 90 days, showing no significant association, although point estimates suggested a higher risk in the no-reflow group (RR 2.04, 95% CI 0.73–5.65; *I*^2^ = 70.0%; Figure 4(b)).

#### Haemorrhagic complications

**Figure 5.**
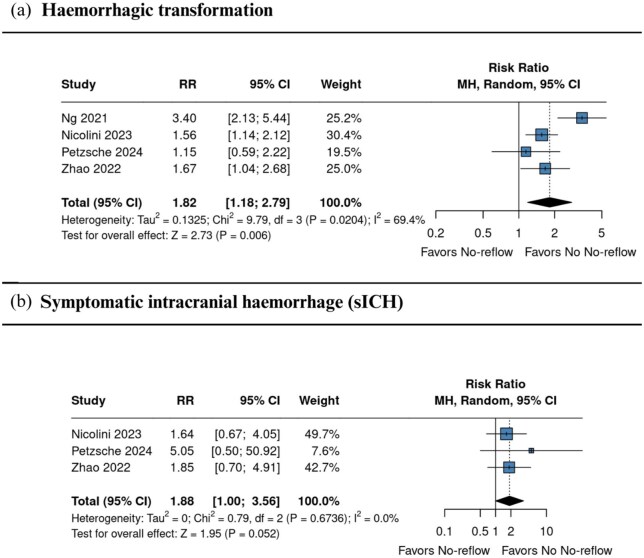
Comparison between no reflow and no no-reflow, in terms of haemorrhagic transformation (a), and Mortality at 90 days (b) in patients with and without the no-reflow phenomenon. Effect estimates are expressed as RRs with 95% confidence intervals, weighted by study precision. CI: confidence interval; RR: risk ratio.

Four studies^1,6,22,24^ reported haemorrhagic transformation, which was more frequent in the no-reflow group (RR 1.82, 95% CI 1.18–2.79; *I*^2^ = 69.4%; Figure 5(a)). Three studies^1,22,24^ reported sICH, with a borderline non-significant higher risk among patients with no-reflow (RR 1.88, 95% CI 1.00–3.56; *I*^2^ = 0.0%; Figure 5(b)). Definitions of sICH varied slightly but consistently required radiographic haemorrhage associated with neurological deterioration, most often defined as a ⩾4-point increase in NIHSS score (Table S1).

#### Reperfusion outcomes

Four studies^1,22–24^ reported successful reperfusion (eTICI 2c–3), showing a higher rate in patients without no-reflow (RR 1.74 (95% CI 1.01–3.00); *p* = 0.046; *I*^2^ = 97.4%; Figure S1). For eTICI 2b–3,^1,22–24^ no statistically significant difference was observed between groups (RR 1.09 (95% CI 0.98–1.20); *p* = 0.097; *I*^2^ = 92.9%; Figure S2).

#### Neurological severity and lesion volume

Two studies^1,23^ evaluated NIHSS scores at 24 h, with no significant difference between groups (MD 3.40 (95% CI −2.19 to 8.98); *p* = 0.233; *I*^2^ = 90.5%; Figure S5). Follow-up infarct volume^1,23^ was also similar (MD 5.79 mL (95% CI −8.68 to 20.26); *p* = 0.433; *I*^2^ = 89.1%; Figure S6).

#### Sensitivity analyses and subgroup analysis

To assess the robustness of the findings and explore potential sources of heterogeneity, leave-one-out sensitivity analyses were performed for all primary outcomes. For functional independence (mRS 0–2), exclusion of Nicolini et al.^1^ reduced heterogeneity from 68.7% to 25.1% (RR 0.60 (95% CI 0.32–1.10)). For haemorrhagic transformation, Ng et al.^6^ was the most influential study, with its exclusion reducing heterogeneity from 60.5% to 0.0% (RR 1.52 (95% CI 1.20–1.94)). Regarding successful reperfusion, the removal of Rivet et al.^25^ yielded the lowest heterogeneity for eTICI 2c–3 (*I*^2^ = 0.0%; RR 1.38 (95% CI 1.27–1.49)), while exclusion of Rivet et al.^25^ yielded the lowest heterogeneity for eTICI 2b–3 (*I*^2^ = 0.0%; RR 1.38 (95% CI 1.27–1.49)). For all-cause mortality at 90 days, the lowest heterogeneity was observed upon excluding Nicolini et al.,^1^ which reduced *I*^2^ to 7.7% (RR 3.15 (95% CI 1.36–7.32)). All corresponding leave-one-out sensitivity analyses are shown in Figures S17–S28.

Subanalyses based on study design provided in Figures S3–S16. In the subgroup analysis restricted to two post-hoc RCTs (Ng et al.^6^ and Nicolini et al.^1^), follow-up lesion volume was significantly greater in patients with no-reflow (MD 12.70 mL (95% CI 8.46–16.94)), contrasting with the overall analysis, which showed no significant difference (MD 5.79 mL (95% CI −8.68 to 20.26); *p* = 0.433). Between-group heterogeneity was significant (χ^2^ = 9.20; *p* = 0.0024), indicating that results from post-hoc RCTs may differ from the broader evidence base. These findings underscore the need for prospective trials designed to evaluate the tissue-level impact of no-reflow.

#### Quality assessment

Figures S29 and S30 present the risk-of-bias assessment summary. All included studies were judged to have either low or moderate overall risk of bias, except for Ter Schiphorst et al.,^20^ which was rated at critical risk due to serious concerns in confounding (Domain 1). Among the randomized trials, Ng et al.^6^ was assessed as low risk across all domains, while Rivet et al.^25^ showed some concerns in deviations from intended interventions (Domain 3) and in the selection of the reported result (Domain 5). In the non-randomized studies, confounding (Domain 1) was the most frequent source of bias, with serious ratings observed in Ng et al.^6^ and Ter Schiphorst et al.^20^ Overall, methodological concerns were concentrated in domains related to confounding, classification of interventions, and missing data. Visual inspection of the funnel plot did not reveal asymmetry, suggesting that publication bias may not be present (Figure S31-S36). However, the analysis of funnel plots with fewer than <10 studies has limited accuracy.^13^

## Discussion

This systematic review and meta-analysis included eight studies and aimed to clarify the prevalence and clinical impact of the no-reflow phenomenon in patients with AIS treated with EVT. The primary objective was to evaluate the association of no-reflow phenomenon with key outcomes, including functional outcomes, mortality, hemorrhagic transformation, and early neurological deterioration. Our findings suggest that the presence of no-reflow phenomenon is associated with a reduced likelihood of early neurological recovery and functional independence, as well as an increased risk of sICH and hemorrhagic transformation despite similar successful reperfusion rates compared to no no-reflow group.

Subgroup analyses by study design showed that no-reflow had a greater impact in RCTs, with reduced reperfusion, larger infarcts, worse early NIHSS, and lower rates of excellent outcome (mRS 0–1). Early recovery and hemorrhagic transformation were affected in both designs, but more strongly in RCTs. These differences highlight the influence of study design on outcome estimates. Prior studies have reported unfavorable outcomes in patients with no-reflow when compared with patients with complete angiographic recanalization and tissue reperfusion.^5^ In line, our systematic review and meta-analysis revealed that the no-reflow phenomenon is associated with trends toward worse clinical outcomes in patients with AIS treated with EVT. Although several comparisons did not reach statistical significance, comparable direction of the association between poorer neurological and functional outcomes emerged in the no-reflow phenomenon group.

The likelihood of achieving excellent functional outcome (mRS 0–1) was lower in the no-reflow phenomenon group (RR 0.64, 95% CI 0.17–2.39; *I*^2^ = 92.5%), as was the probability of achieving functional independence (mRS 0–2) (RR 0.78, 95% CI 0.42–1.29; *I*^2^ = 68.7%). These findings indicate a potential negative impact of no-reflow phenomenon on long-term recovery. Notably, early neurological recovery was significantly reduced among patients with no-reflow phenomenon (RR 0.76, 95% CI 0.64–0.90; *I*^2^ = 0.0%), highlighting an early effect of this microvascular phenomenon on post-procedural recovery.

Patients with no-reflow phenomenon had significantly higher NIHSS scores at 24 h (MD 3.40, 95% CI −2.19 to 8.98; *I*^2^ = 90.5%), reinforcing the association with worse short-term neurological outcomes. Luijten et al.^26^ used Pseudo-continuous ASL imaging to examine the relationship between cerebral perfusion and neurological status at 24 h in a cohort of 44 patients, 40 of whom underwent mechanical thrombectomy.^26^ They found that higher relative cerebral blood flow within the infarct core was associated with lower NIHSS scores at 24 h, suggesting that better microvascular perfusion may contribute to improved early neurological outcomes.^26^

Regarding imaging markers, our findings are in line with previous reports indicating that patients with no-reflow tend to have larger infarct volumes.^6^ Specifically, we observed a greater baseline lesion volume in the no-reflow phenomenon group (MD 5.79 mL, 95% CI −8.68 to 20.26; *I*^2^ = 89.1%), supporting the notion that impaired microvascular perfusion may contribute to more extensive tissue injury. Significant differences in successful reperfusion rates between groups were observed when using eTICI 2c–3 (RR 1.74, 95% CI 1.01–3.00; *I*^2^ = 97.4%), whereas no statistically significant difference was found with eTICI 2b–3 (RR 1.09, 95% CI 0.98–1.20; *I*^2^ = 92.9%), suggesting that angiographic reperfusion may not reliably reflect microvascular perfusion status. This apparent discrepancy may be explained by limitations in angiographic assessment, as operators can overestimate reperfusion success based on DSA alone.^27^ Moreover, digital subtraction angiography does not reliably capture microvascular flow disturbances, such as the no-reflow phenomenon, which may be better detected through advanced perfusion imaging techniques.^28^ The study by Mujanovic et al. highlights that patients exhibiting the no-reflow phenomenon experienced worse clinical outcomes despite achieving optimal angiographic reperfusion (eTICI 2c–3), when compared to those with partial recanalization (eTICI 2b). Their outcomes were as poor as those of patients in whom thrombectomy failed.^5^

The pooled prevalence of no-reflow phenomenon was 20.5% (95% CI 6.2–49.9; *I*^2^ = 96.9%), indicating a relatively frequent occurrence post-EVT. This finding is comparable to previously reported rates, such as those described by Mujanovic et al.,^11^ who observed similar prevalence, reinforcing the consistency of no-reflow phenomenon as a common complication following EVT.^11^ Importantly, the risk of sICH was significantly higher in the no-reflow phenomenon group (RR 1.88, 95% CI 1.00–3.56; *I*^2^ = 0.0%), as was the risk of hemorrhagic transformation (RR 1.82, 95% CI 1.18–2.79; *I*^2^ = 69.4%). Mortality at 90 days was also numerically higher among patients with no-reflow phenomenon (RR 2.04, 95% CI 0.73–5.65; *I*^2^ = 70.0%), indicating a potential trend toward increased risk that warrants further investigation in larger studies.

Taken together, these findings suggest that no-reflow phenomenon may exert clinically meaningful effects on early and potentially long-term outcomes, despite similar rates of macrovascular reperfusion. The substantial heterogeneity and limited number of studies underscore the need for further standardized definition of no-reflow to validate these associations and guide targeted interventions.

The absence of a standardized definition for the no-reflow phenomenon represents one of the main limitations of this meta-analysis and of any future attempt to investigate this topic. According to the original definition, no-reflow is characterized as a patchy phenomenon, with small regions of absent or reduced blood flow in areas of brain tissue with preserved macrovascular perfusion.^29^ However, the included studies adopted dichotomous outcomes (present/absent) without clear volumetric or functional criteria, which compromises comparability across findings. In summary, it remains unclear which perfusion maps are best suited to detect the no-reflow phenomenon. This is reflected in the variety of definitions used across the included studies and highlights the need to establish consensus criteria. Moreover, the evolution of tissue reperfusion after intervention is a dynamic process influenced by complex mechanisms at both the macrovascular and microvascular levels, which are not always captured by conventional angiographic techniques.^10^ Perfusion imaging has proven to be more sensitive for detecting microvascular alterations, and therefore represents a promising tool for refining the characterization of no-reflow.^25^ Recently, a new approach has been proposed to perform perfusion imaging directly in the angiography suite, using technologies such as flat-panel computed tomography (flat-panel CT), which could allow real-time assessment of tissue status during the endovascular procedure.^5^ These methodological advances may help develop more consistent and standardized definitions, which are essential for research progress and for the clinical application of the no-reflow concept.

### Limitation

This study has several limitations that warrant consideration. First, the number of included studies was relatively small, which limits the overall statistical power and the generalizability of the findings. Additionally, many of the pooled analyses were based on a limited number of studies, and several outcomes exhibited high heterogeneity, suggesting considerable variability in study design, patient populations, definitions of no-reflow, and outcome assessment. However, despite these disparities, present results on the association between no-reflow and poor outcomes are in line with previously published work on this topic. The diagnosis of no-reflow phenomenon was not standardized across studies, with variations in imaging modalities and timing, potentially introducing bias or misclassification. Furthermore, the observational nature of the included studies precludes causal inference, and residual confounding cannot be ruled out. Finally, the wide confidence intervals in several estimates reflect imprecision and underscore the need for larger and methodologically rigorous studies to better define the clinical implications of no-reflow phenomenon in AIS patients undergoing EVT.

## Conclusion

This meta-analysis suggests that no-reflow phenomenon may be relatively common after EVT for AIS and potentially associated with poorer early neurological recovery, reduced rates of functional independence, and increased risks of sICH and hemorrhagic transformation. Despite high heterogeneity and small sample sizes, the direction of associations was generally consistent across outcomes. These preliminary results underline the need to improve diagnostic consistency and support the rationale for well-designed, prospective studies to further clarify the clinical implications of no-reflow phenomenon in this setting.

## Supplementary Material

ds-eso_23969873251376846

## Registration PROSPERO protocol number

CRD420251015325.

## Data availability statement

All data included in this review are available in the published articles referenced in the manuscript.
