# Supplementary material for: Association between the no-reflow phenomenon and clinical outcomes after endovascular treatment for acute ischemic stroke: A systematic review and meta-analysis
Source: Eur Stroke J. 2026 Jan 1;11(1):23969873251376846. doi: 10.1093/esj/23969873251376846 (PMC12866224; doi:10.1093/esj/23969873251376846)
Supplement: ds-eso_23969873251376846 [file ds-eso_23969873251376846.zip › sj-docx-1-eso-10.1177_23969873251376846.docx]

**Supplementary Material**

**Supplementary Figures**

**1. Reperfusion Outcomes**

**1.1 Figure S1.** Successful reperfusion defined as eTICI 2c–3 in patients with and without no-reflow.

**1.2 Figure S2.** Successful reperfusion defined as eTICI 2b–3 in patients with and without no-reflow.

**1.3 Figure S3.** Successful reperfusion defined as eTICI 2b–3 in patients with and without no-reflow divided by study design.

**1.4 Figure S4.** Successful reperfusion defined as eTICI 2c–3 in patients with and without no-reflow divided by study design.

**2. Clinical Outcomes**

**2.1 Figure S5.** NIHSS scores at 24 hours in patients with and without no-reflow.

**2.2 Figure S6.** Follow-up lesion volume (mL) in patients with and without no-reflow.

**2.3 Figure S7.** Follow-up lesion volume (mL) in patients with and without no-reflow divided by study design.

**2.4 Figure S8.** Death at 90 days in patients with and without no-reflow.

**2.5 Figure S9.** Early neurologic recovery in patients with and without no-reflow divided by study design.

**2.6 Figure S10.** Excellent functional outcome (mRS 0-1) at 90 days in patients with and without no-reflow divided by study design.

**2.7 Figure S11.** Functional independence outcome (mRS 0-2) at 90 days in patients with and without no-reflow divided by study design.

**2.8 Figure S12.** Variation of mRS at 90 days in patients with and without no-reflow divided by study design.

**2.9 Figure S13.** Poor outcome (mRS 3-6) at 90 days in patients with and without no-reflow divided by study design.

**2.10 Figure S14.** NIHSS scores at 24 hours in patients with and without no-reflow divided by study design.

**2.11 Figure S15.** Haemorrhagic transformation in patients with and without no-reflow divided by study design.

**2.12** **Figure S16.** Prevalence of No-Reflow in the studies divided by study design.

**3. Sensitivity Analyses (Leave-One-Out)**

**3.1 Figure S17.** Leave-one-out sensitivity analysis for functional independence (mRS 0–2).

**3.2 Figure S18.** Leave-one-out sensitivity analysis for excellent functional outcome (mRS 0–1).

**3.3 Figure S19.** Leave-one-out sensitivity analysis for poor functional outcome (mRS 3–6).

**3.4 Figure S20.** Leave-one-out sensitivity analysis for haemorrhagic transformation.

**3.5 Figure S21.** Leave-one-out sensitivity analysis for successful reperfusion (eTICI 2c–3).

**3.6 Figure S22.** Leave-one-out sensitivity analysis for successful reperfusion (eTICI 2b–3).

**3.7 Figure S23.** Leave-one-out sensitivity analysis for all-cause mortality at 90 days.

**3.8** **Figure S24.** Leave-one-out sensitivity analysis for early neurologic recovery.

**3.9 Figure S25.** Leave-one-out sensitivity analysis for mRS variation at 90 days in patients with and without no-reflow.

**3.10 Figure S26**. Leave-one-out sensitivity analysis for NIHSS scores at 24 hours in patients with and without no-reflow.

**3.11Figure S27.** Leave-one-out sensitivity analysis for sICH in patients with and without no-reflow.

**3.12 Figure S28**. Leave-one-out sensitivity analysis for follow-up lesion volume (mL) in patients with and without no-reflow.

**4. Quality assessment**

**4.2 Figure S29.** Risk of Bias Assessment (Traffic Light Plot - ROBINS-I).

**4.1 Figure S30.** Risk of Bias Assessment (Traffic Light Plot - RoB 2.0).

**5. Tables**

**5.1** **Table S1.** Included studies assessing no-reflow after endovascular treatment.

**6. Funnel Plot analysis**

**6.1 Figure S31.** Funnel Plot analysis of the Prevalence of No-Reflow in the studies.

**6.2 Figure S32.** Funnel Plot analysis of Death at 90 days.

**6.3 Figure S33.** Funnel Plot analysis of eTICI 2b-3.

**6.4 Figure S34.** Funnel Plot analysis of eTICI 2c-3.

**6.5 Figure S35.** Funnel Plot analysis of Hemorrhagic Transformation.

**6.6 Figure S36.** Funnel Plot analysis of symptomatic intracerebral hemorrhage (sICH)

**1. Reperfusion Outcomes**


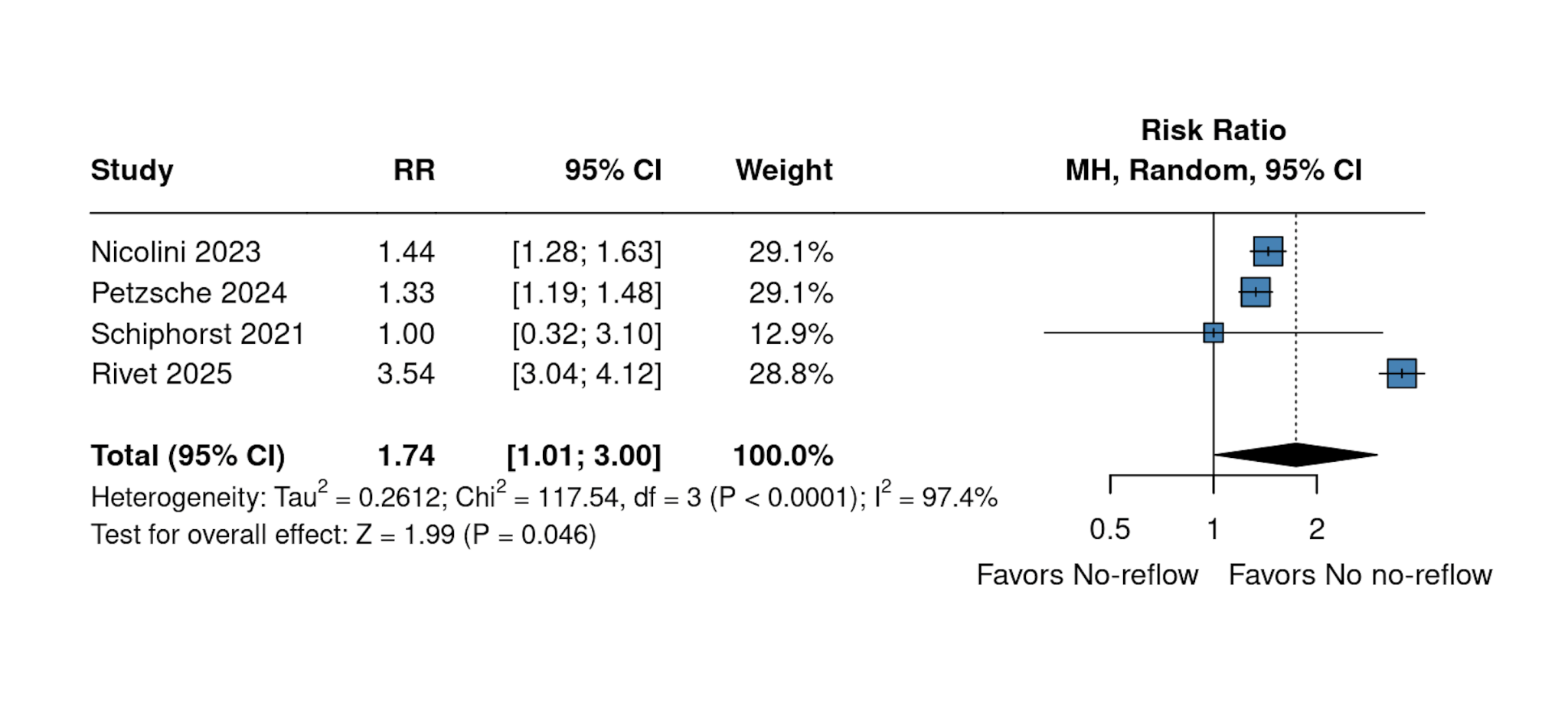


**Figure S1.** Successful reperfusion defined as eTICI 2c–3 in patients with and without no-reflow. CI: Confidence Interval; RR: Risk Ratio; eTICI: Expanded Thrombolysis in Cerebral Infarction.

**
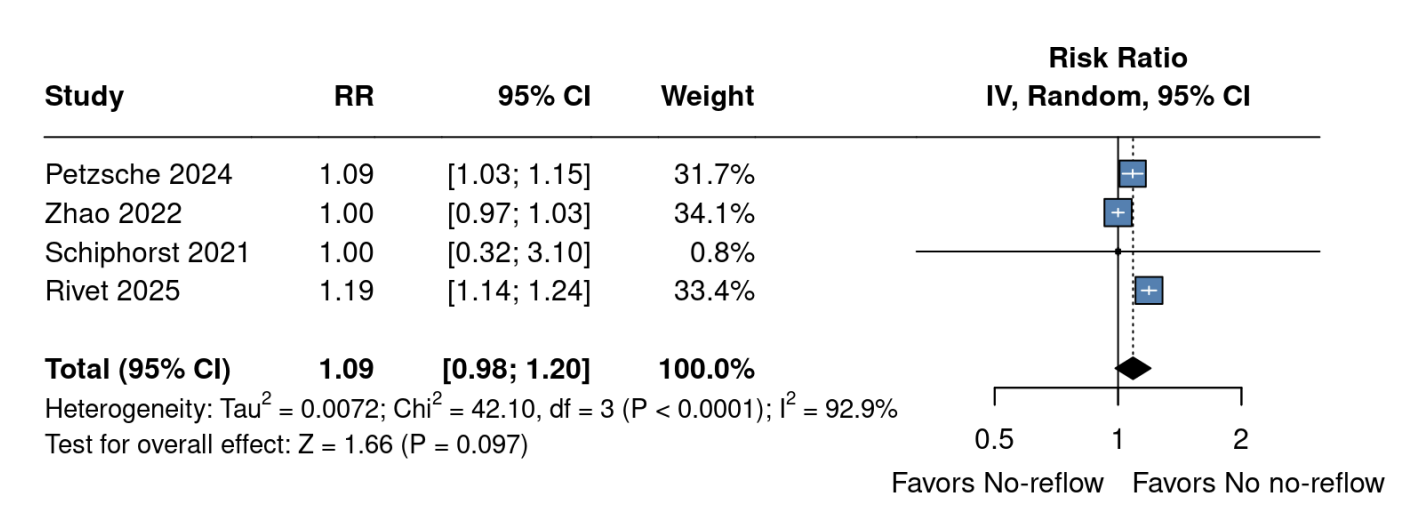
**

**Figure S2.** Successful reperfusion defined as eTICI 2b–3 in patients with and without no-reflow. CI: Confidence Interval; RR: Risk Ratio; eTICI: Expanded Thrombolysis in Cerebral Infarction.


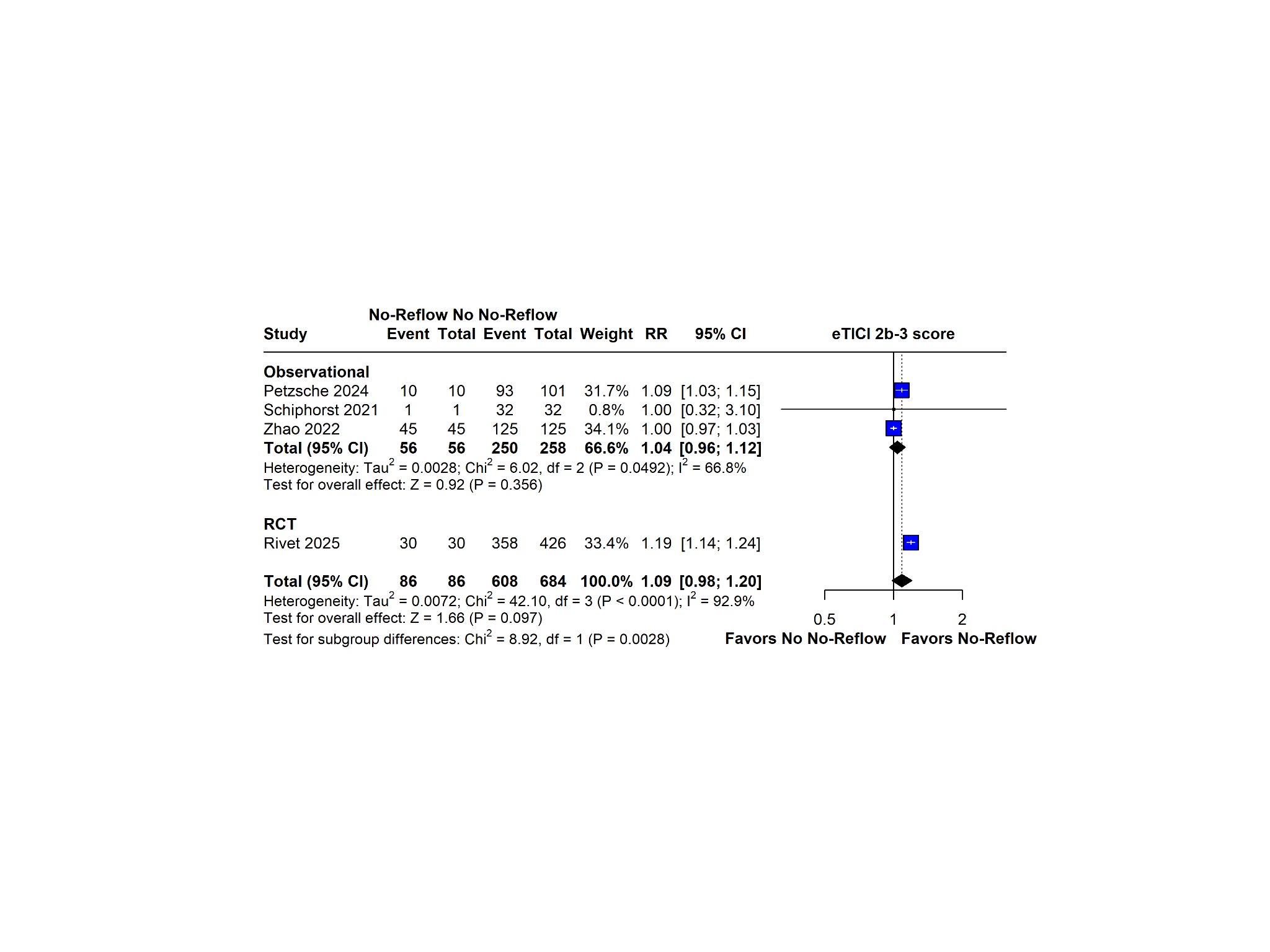


**Figure S3.** Successful reperfusion defined as eTICI 2b–3 in patients with and without no-reflow divided by study design. CI: Confidence Interval; RR: Risk Ratio; eTICI: Expanded Thrombolysis in Cerebral Infarction; RCT: randomized controlled trial.


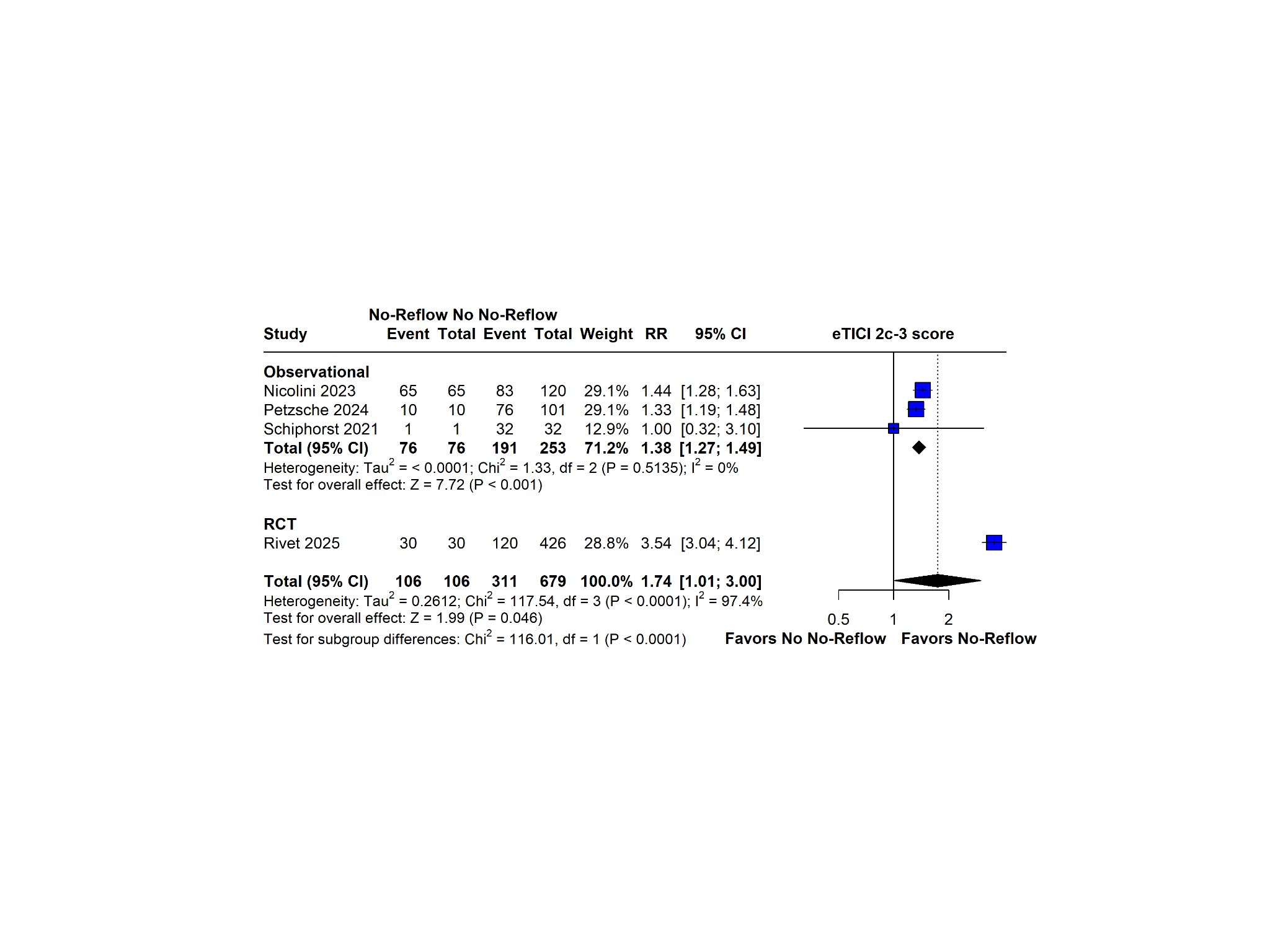


**Figure S4.** Successful reperfusion defined as eTICI 2c–3 in patients with and without no-reflow divided by study design. CI: Confidence Interval; RR: Risk Ratio; eTICI: Expanded Thrombolysis in Cerebral Infarction; RCT: randomized controlled trial.

**2. Clinical Outcomes**

**
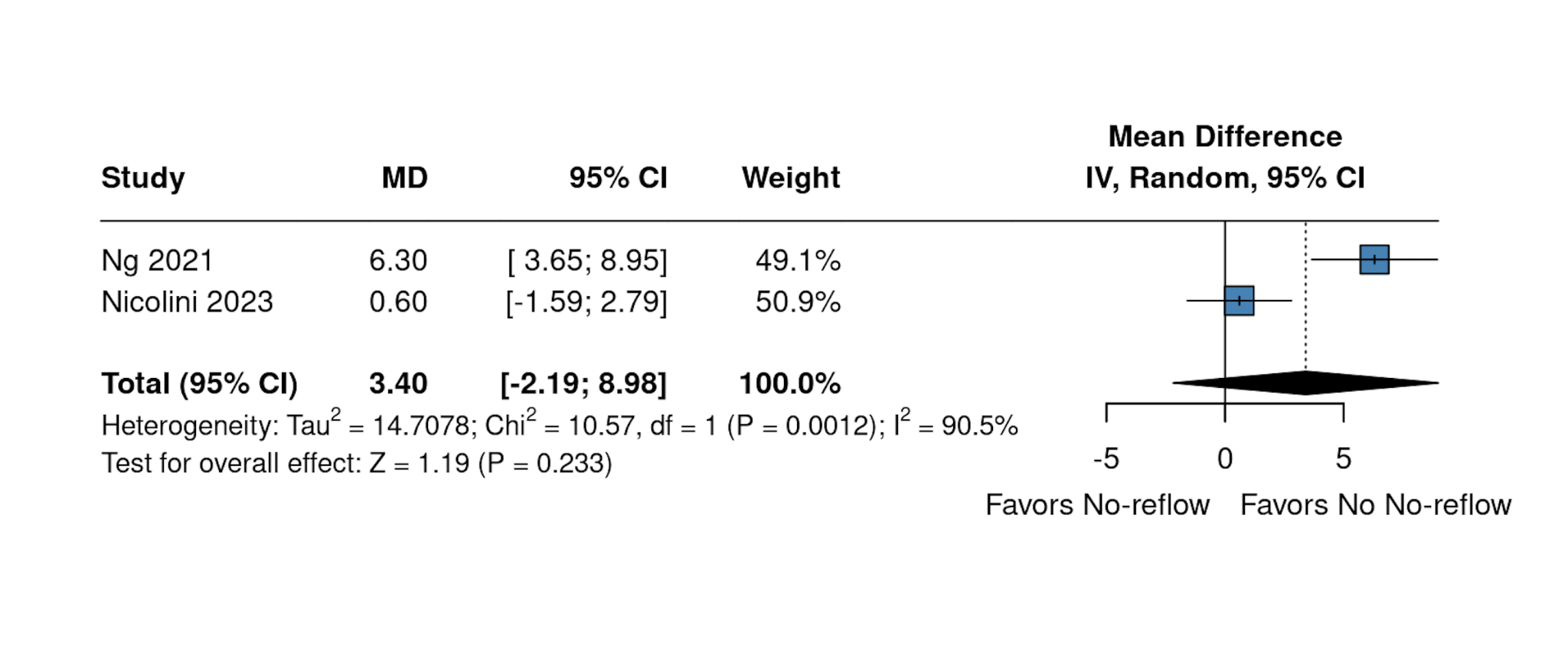
**

**Figure S5.** NIHSS scores at 24 hours in patients with and without no-reflow. CI: Confidence Interval; MD: Mean Difference; NIHSS: National Institutes of Health Stroke Scale.

**
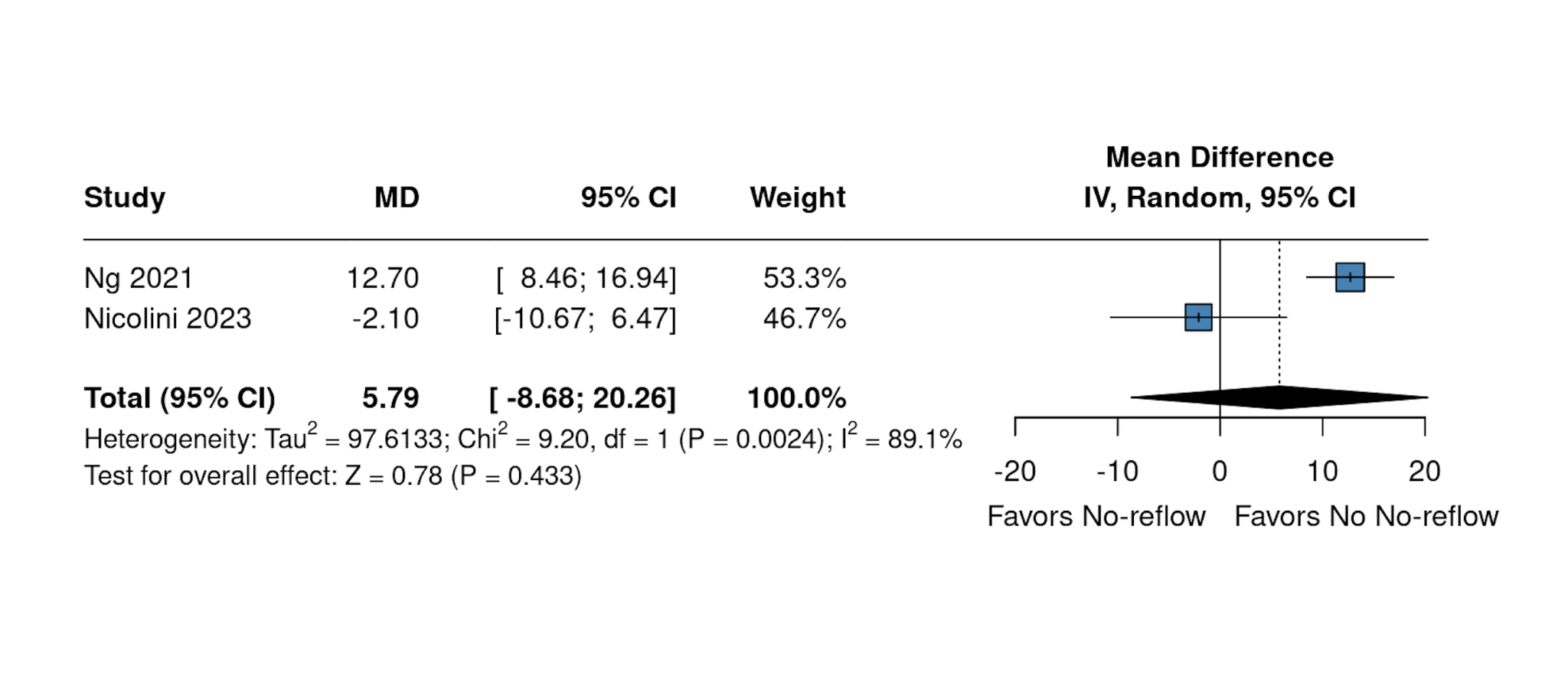
Figure S6.** Follow-up lesion volume (mL) in patients with and without no-reflow. CI: Confidence Interval; MD: Mean Difference.


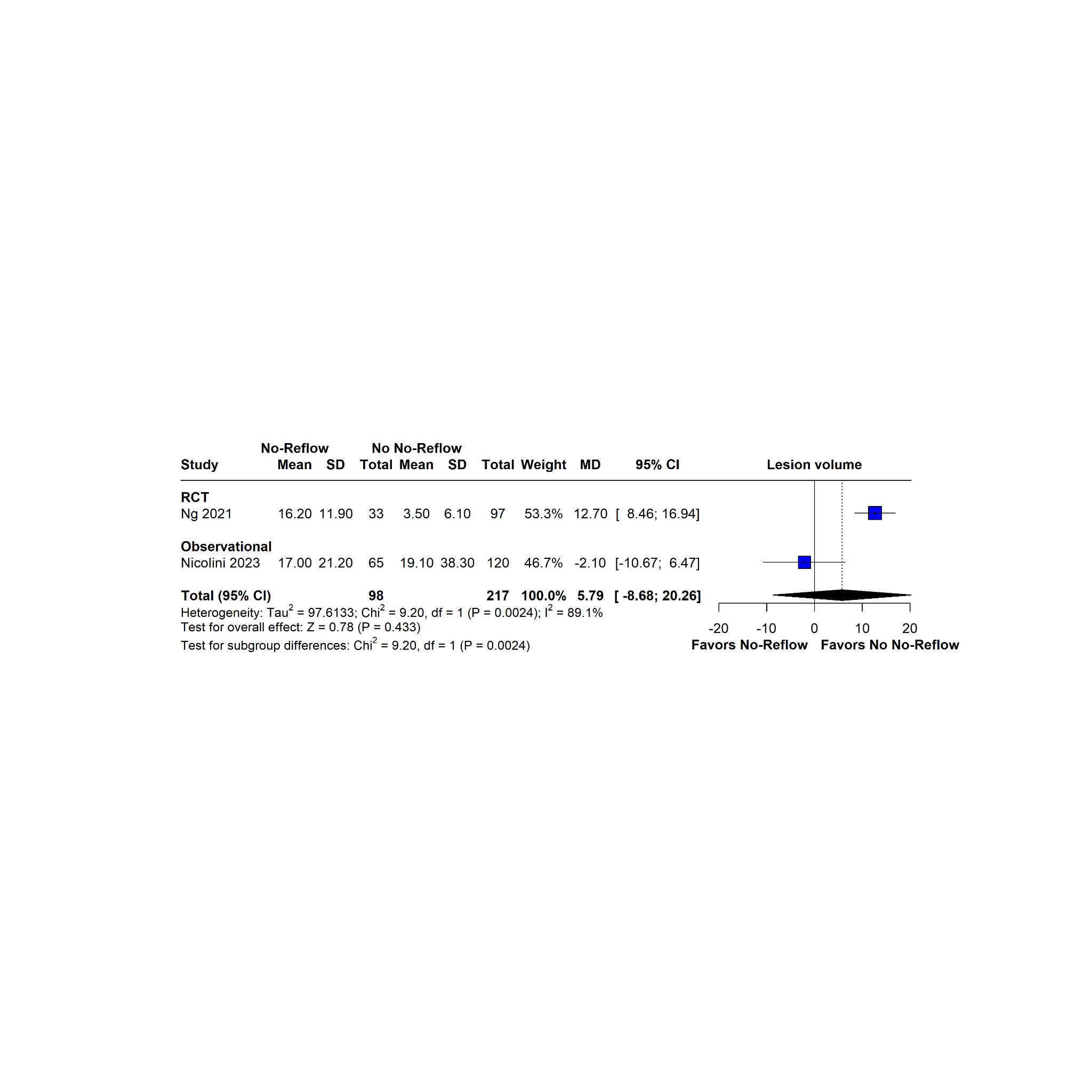


**Figure S7.** Follow-up lesion volume (mL) in patients with and without no-reflow divided by study design. CI: Confidence Interval; MD: Mean Difference; SD: Standard Deviation; RCT: randomized controlled trial.


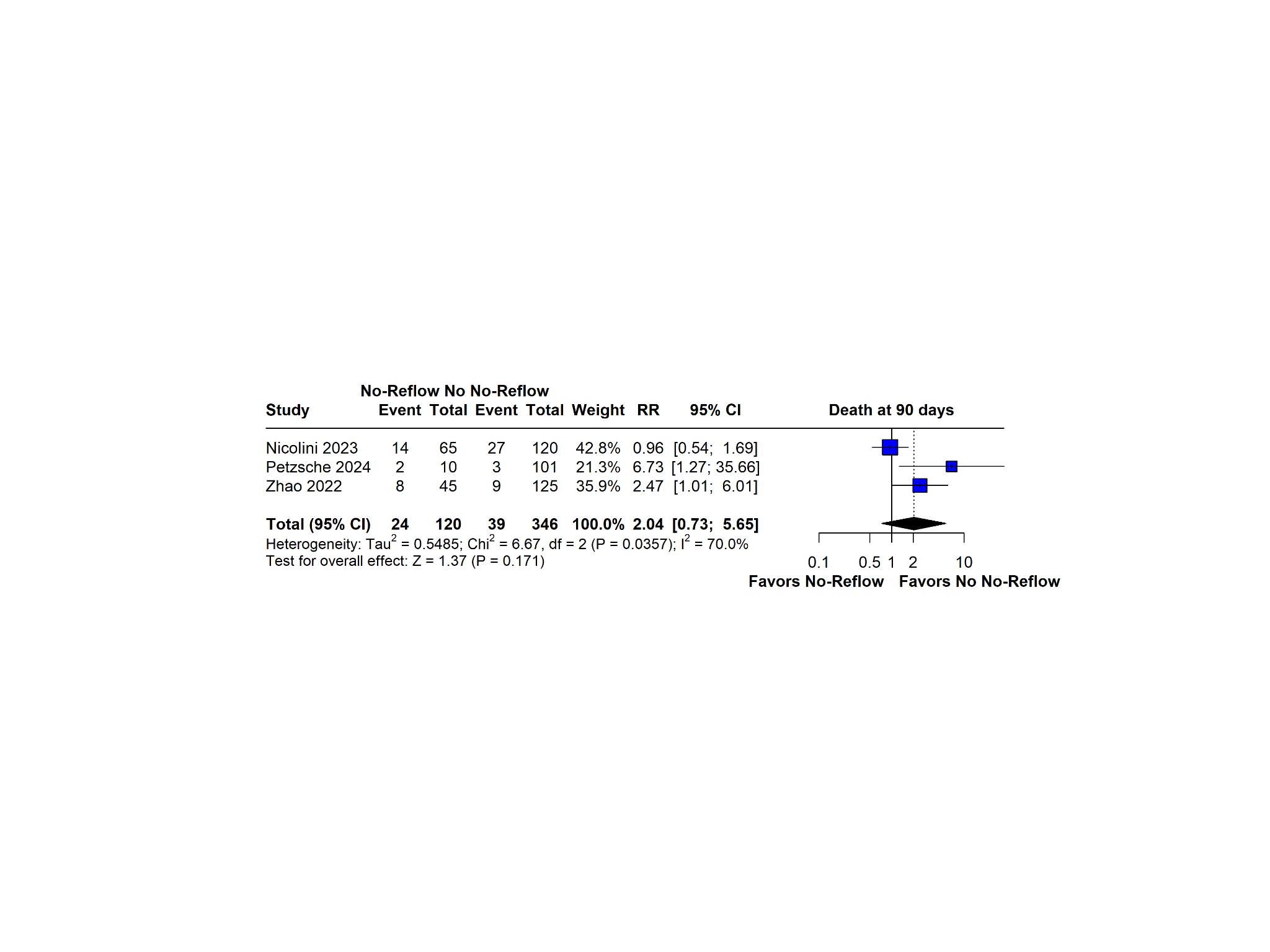


**Figure S8.** Death at 90 days in patients with and without no-reflow. CI: Confidence Interval; RR: Risk Ratio.


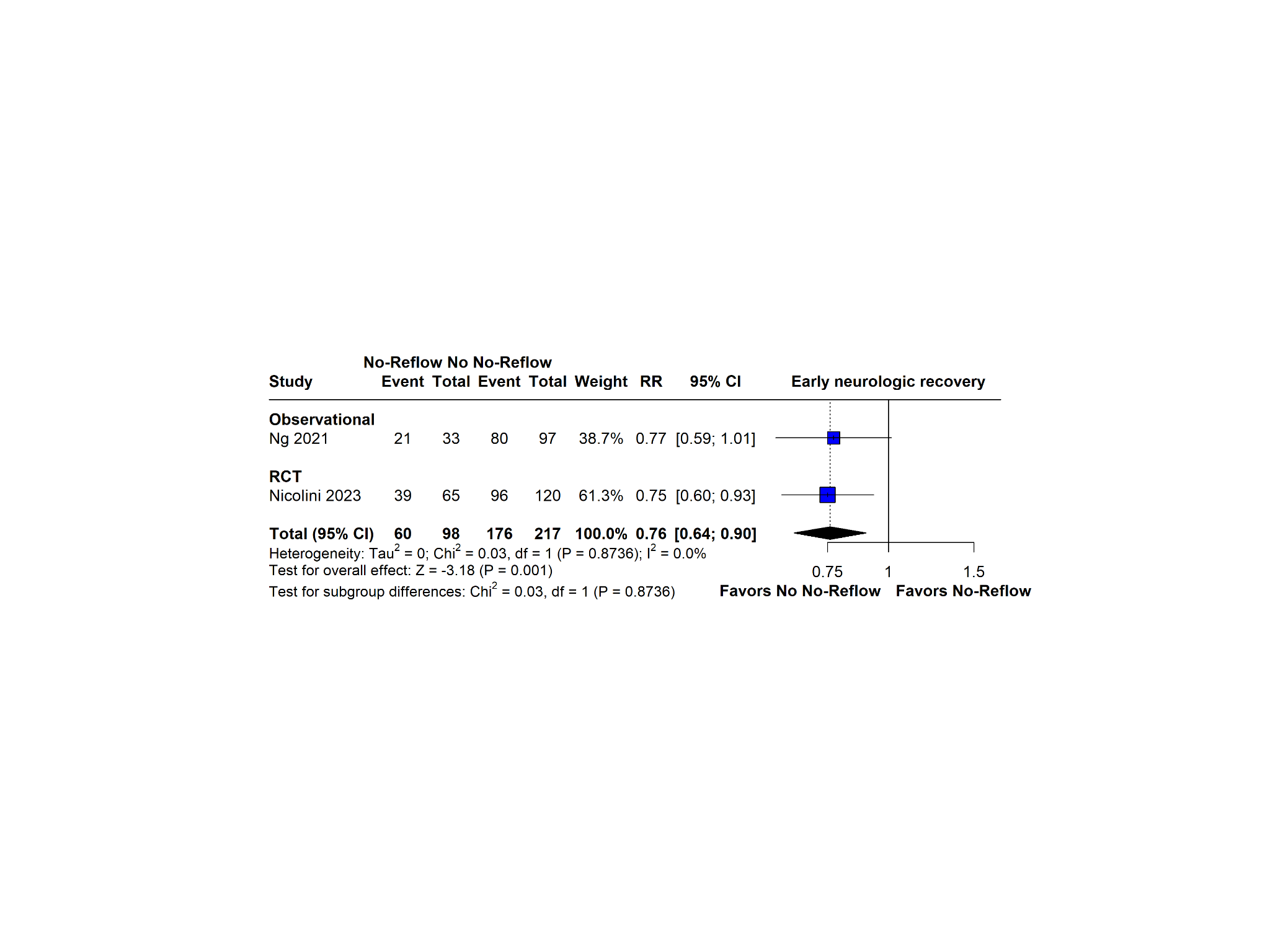


**Figure S9.** Early neurologic recovery in patients with and without no-reflow divided by study design. CI: Confidence Interval; RR: Risk Ratio; RCT: randomized controlled trial.


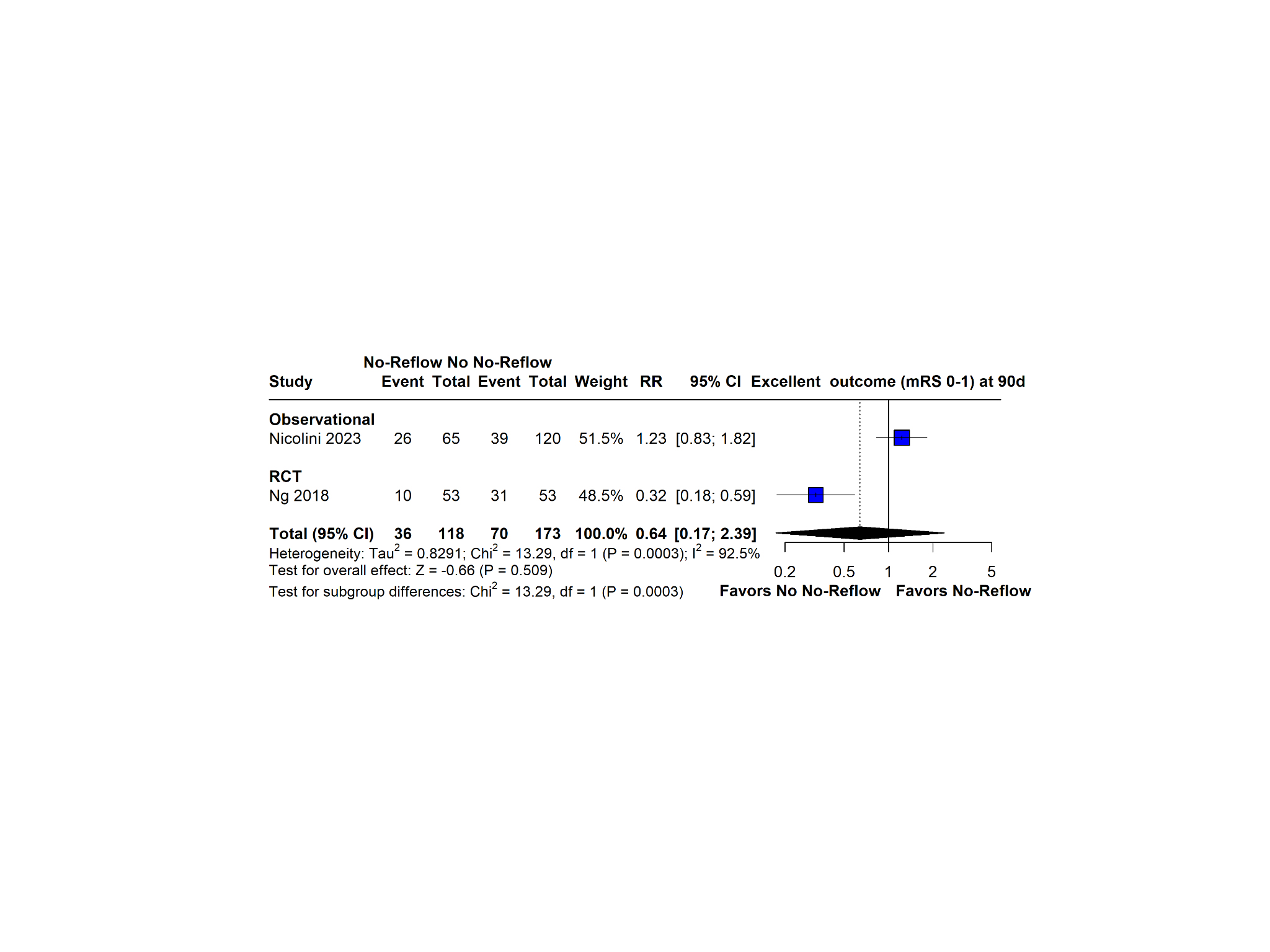


**Figure S10.** Excellent functional outcome (mRS 0-1) at 90 days in patients with and without no-reflow divided by study design. CI: Confidence Interval; RR: Risk Ratio; mRS: modified Rankin Score; RCT: randomized controlled trial.


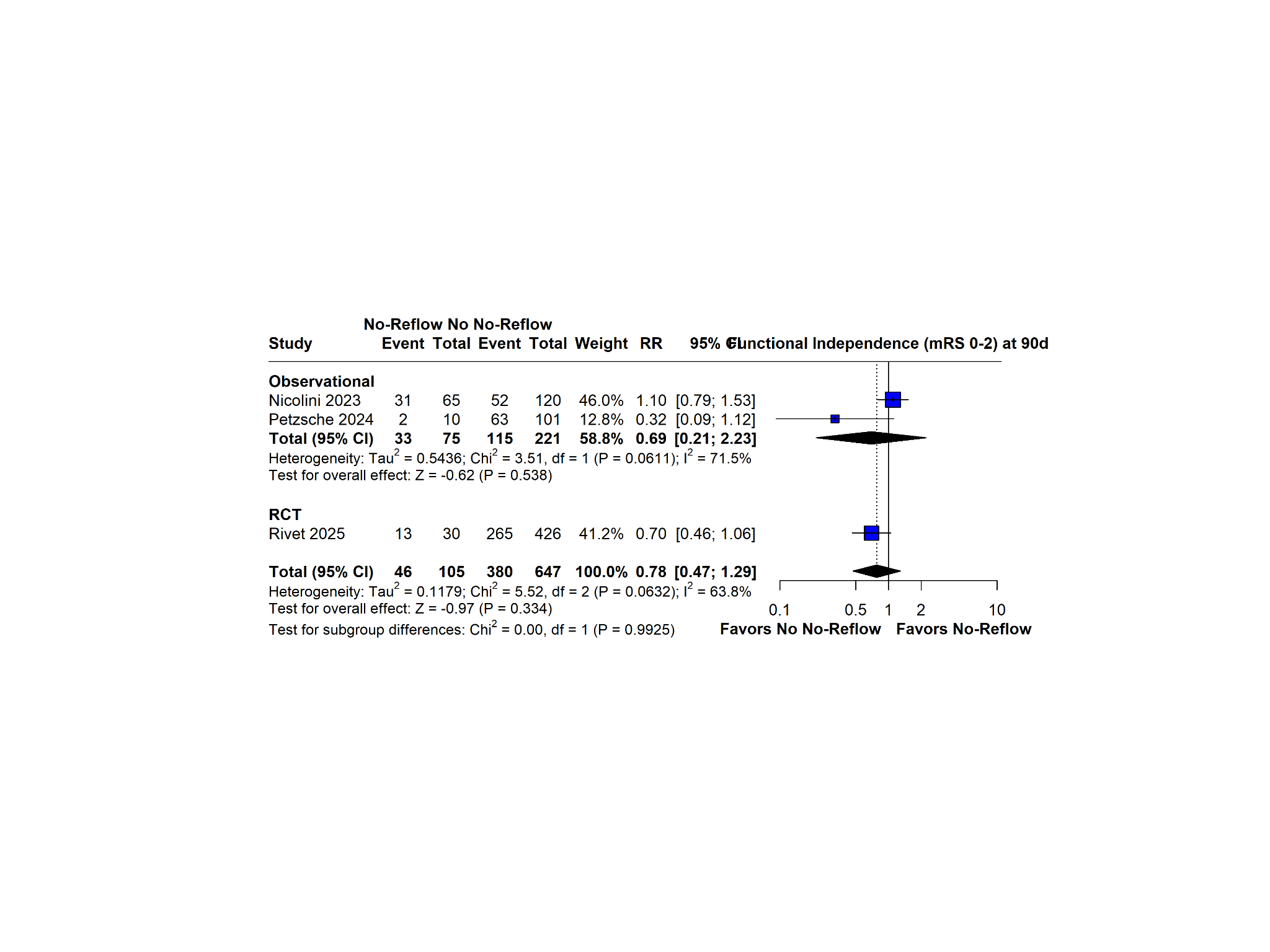


**Figure S11.** Functional independence outcome (mRS 0-2) at 90 days in patients with and without no-reflow divided by study design. CI: Confidence Interval; RR: Risk Ratio; mRS: modified Rankin Score; RCT: randomized controlled trial.


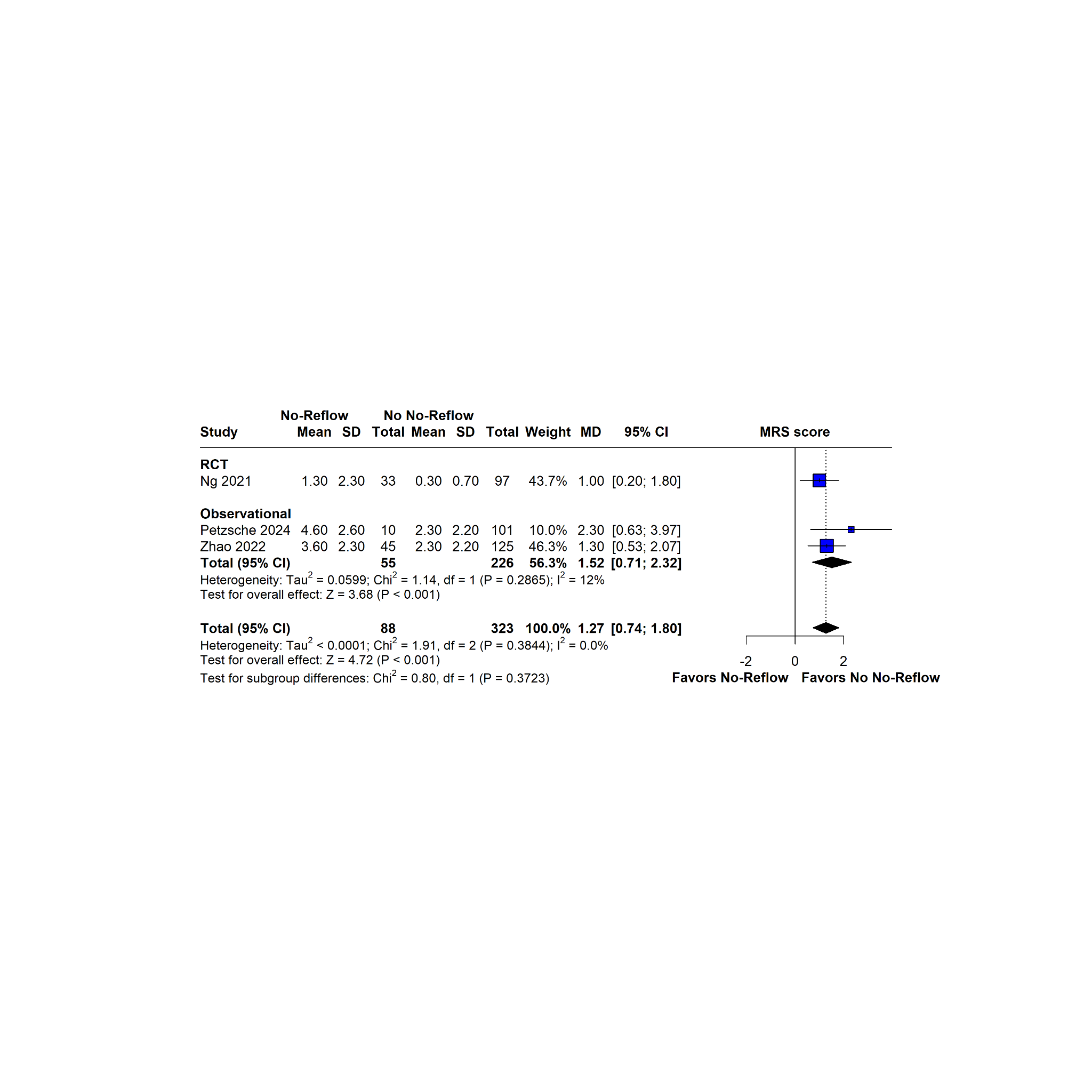


**Figure S12.** Variation of mRS at 90 days in patients with and without no-reflow divided by study design. CI: Confidence Interval; MD: Mean Difference; SD: Standard Deviation; mRS: modified Rankin Score; RCT: randomized controlled trial.


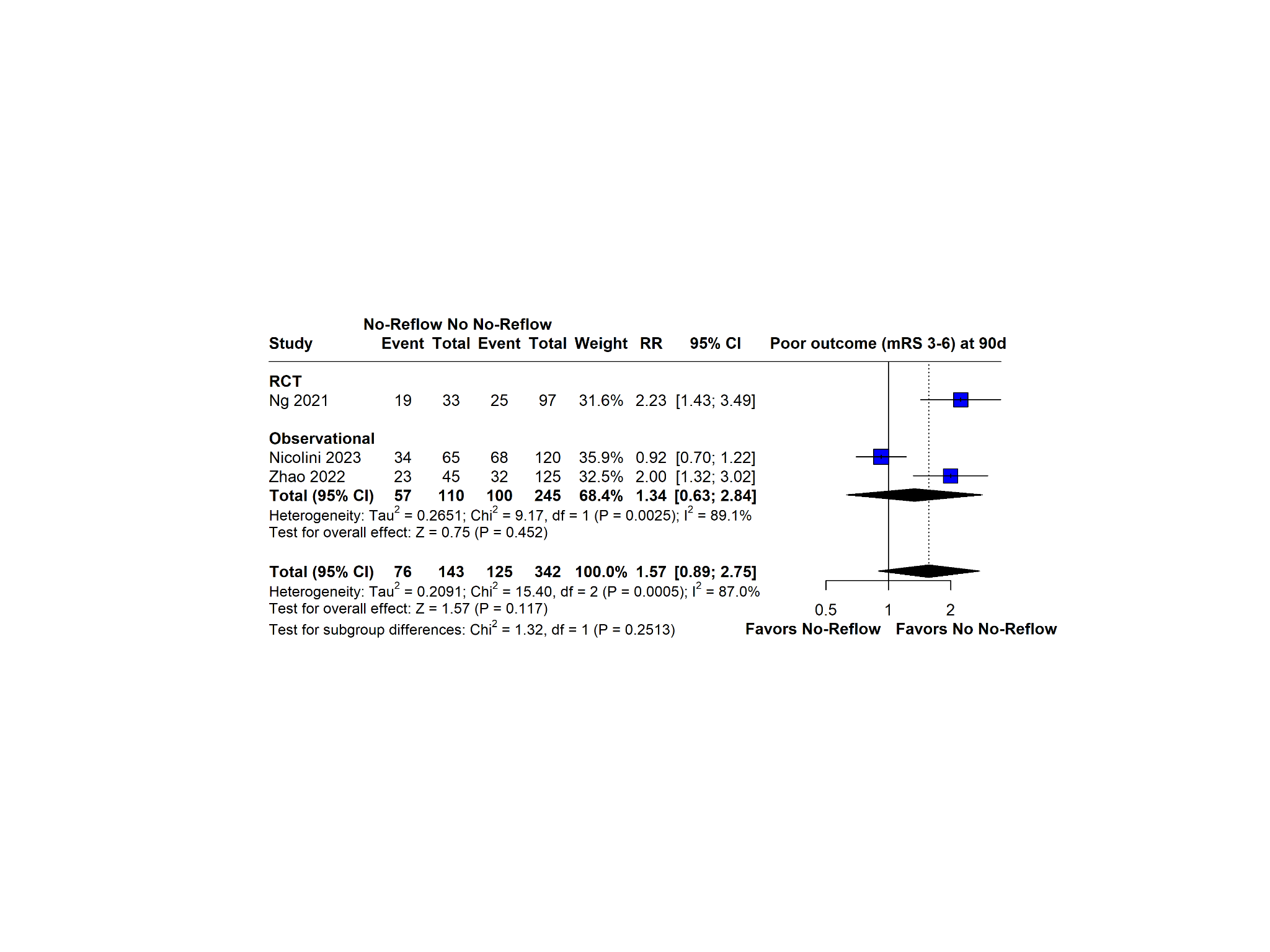


**Figure S13.** Poor outcome (mRS 3-6) at 90 days in patients with and without no-reflow divided by study design. CI: Confidence Interval; RR: Risk Ratio; mRS: modified Rankin Score; RCT: randomized controlled trial..


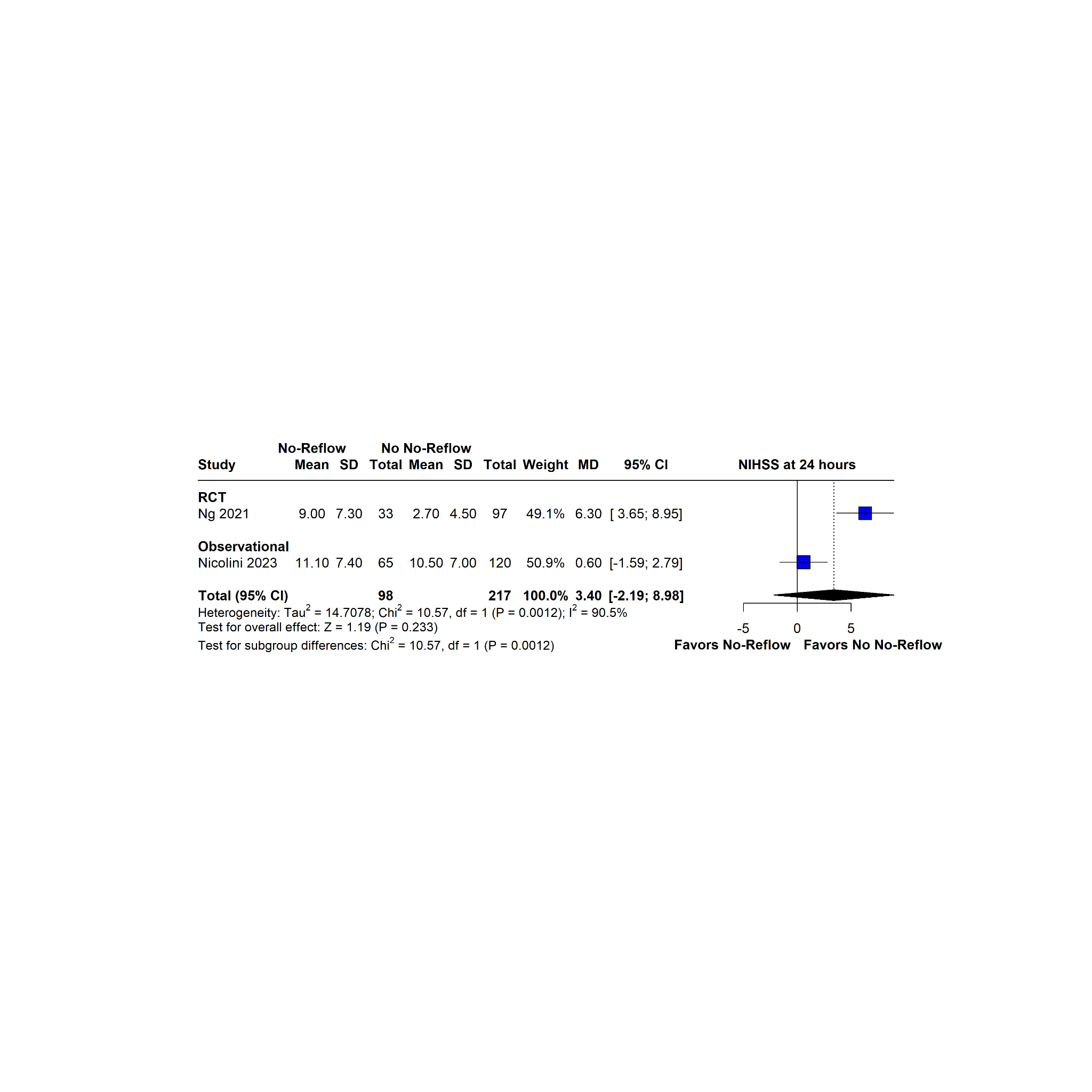


**Figure S14.** NIHSS scores at 24 hours in patients with and without no-reflow divided by study design. CI: Confidence Interval; MD: Mean Difference; NIHSS: National Institutes of Health Stroke Scale; RCT: randomized controlled trial.


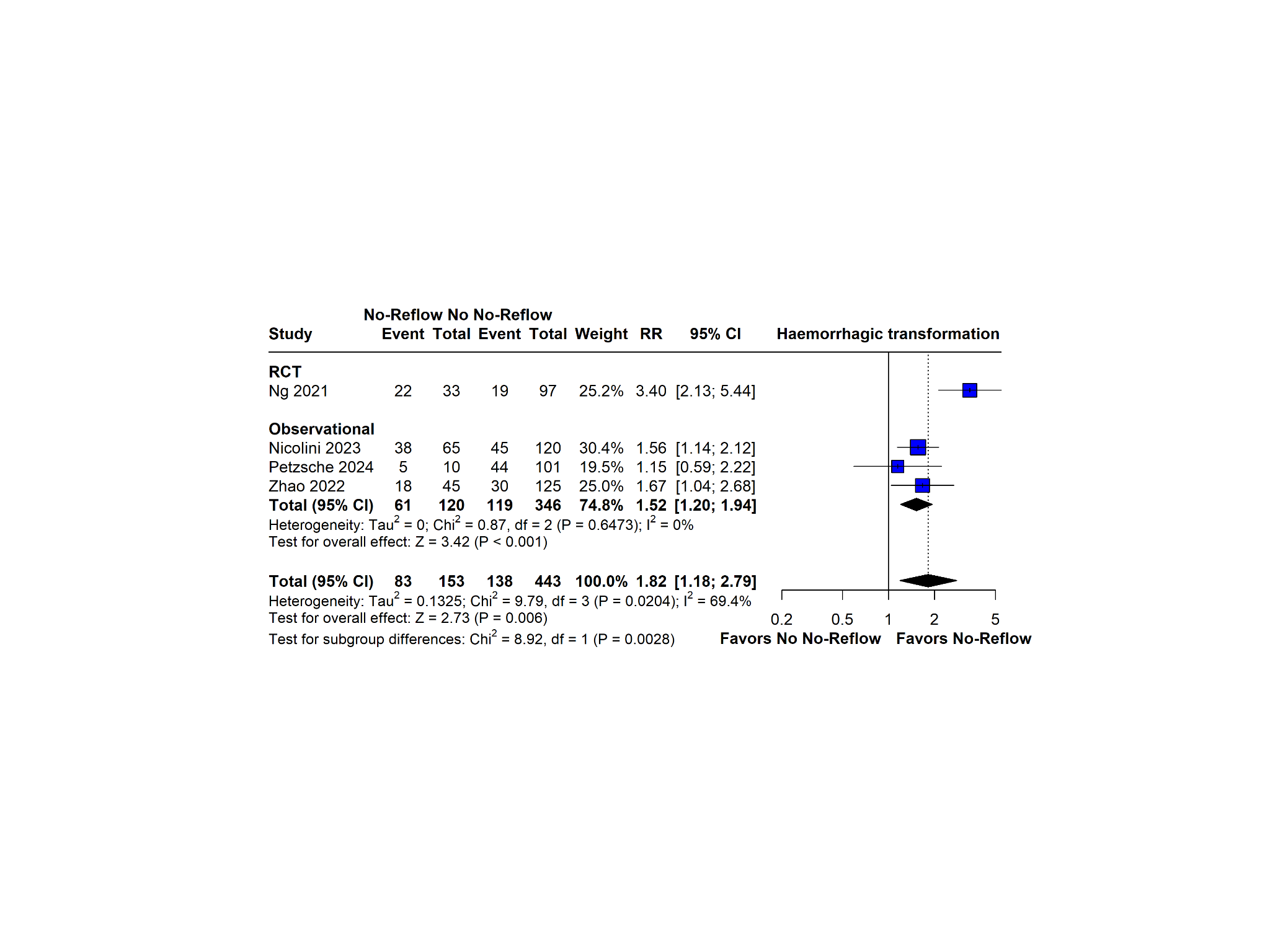


**Figure S15.** Haemorrhagic transformation in patients with and without no-reflow divided by study design. CI: Confidence Interval; RR: Risk Ratio; RCT: randomized controlled trial.


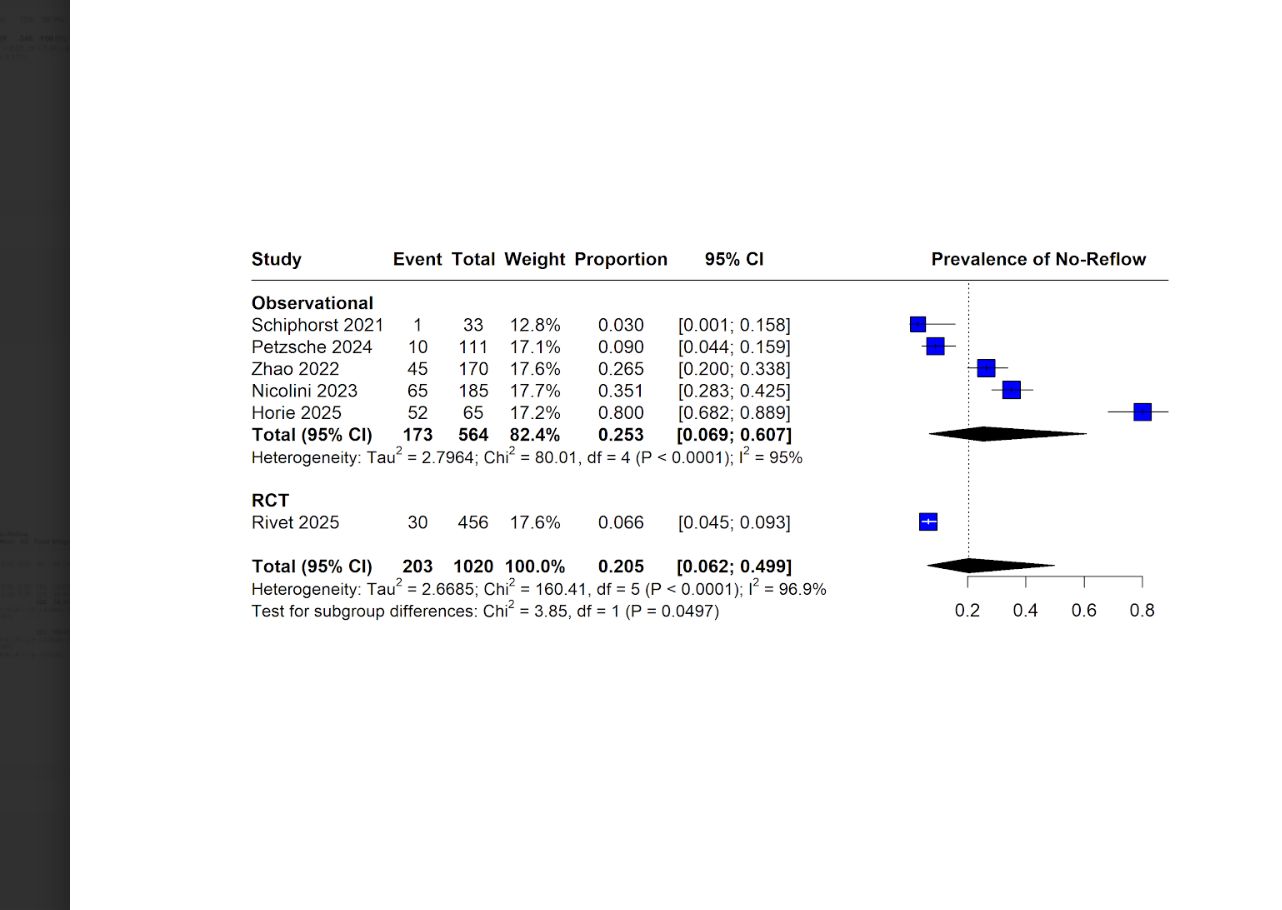


**Figure S16.** Prevalence of No-Reflow in the studies divided by study design. CI: Confidence Interval; RCT: randomized controlled trial.

**3. Sensitivity Analyses (Leave-One-Out)**

**
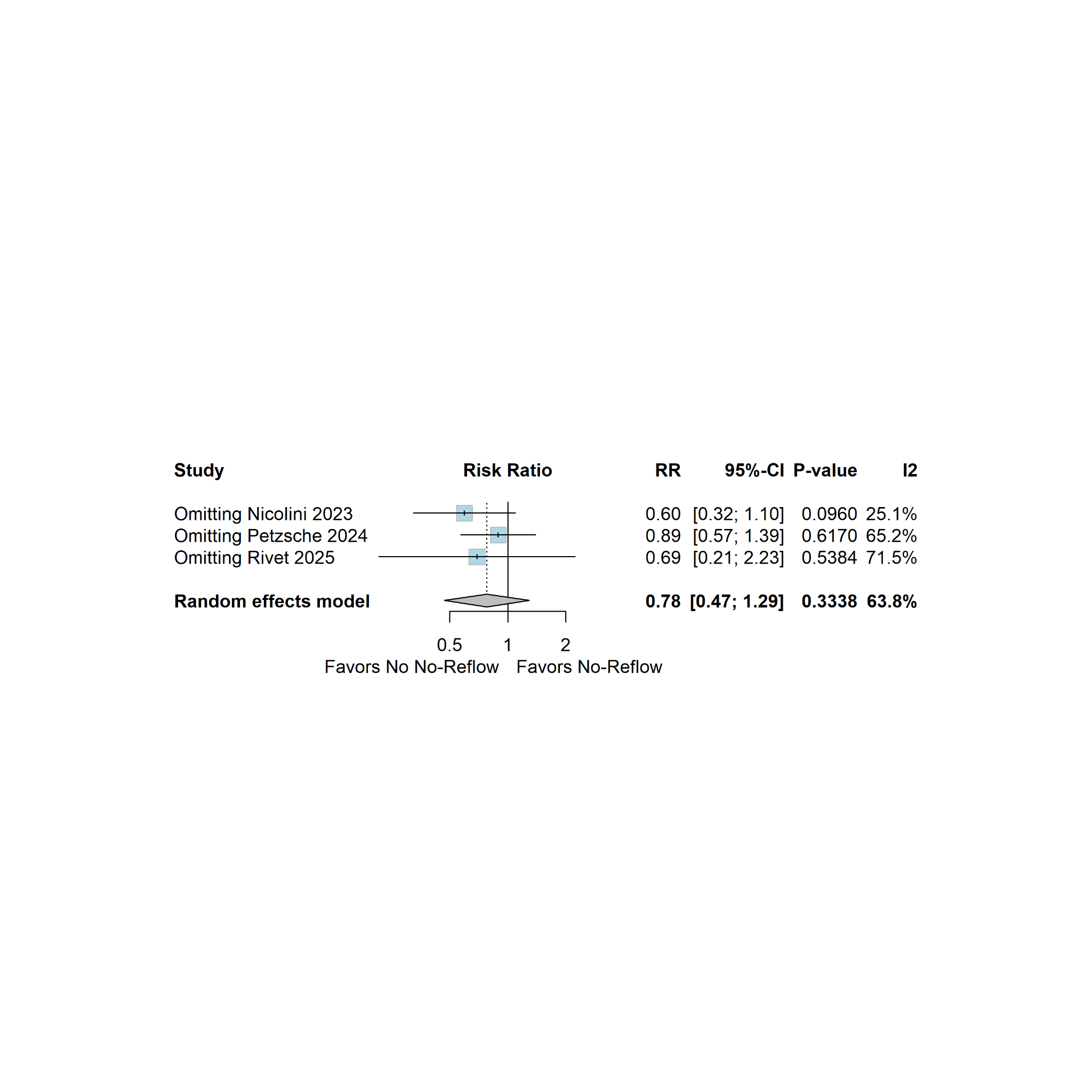
**

**Figure S17.** Leave-one-out sensitivity analysis for functional independence (mRS 0–2). Omitting Nicolini et al. (2023) reduced heterogeneity to 25.1%, yielding a pooled RR of 0.60 (95% CI 0.32–1.10), suggesting moderate influence on the overall estimate. CI: Confidence Interval; RR: Risk Ratio; I²: measure of heterogeneity.


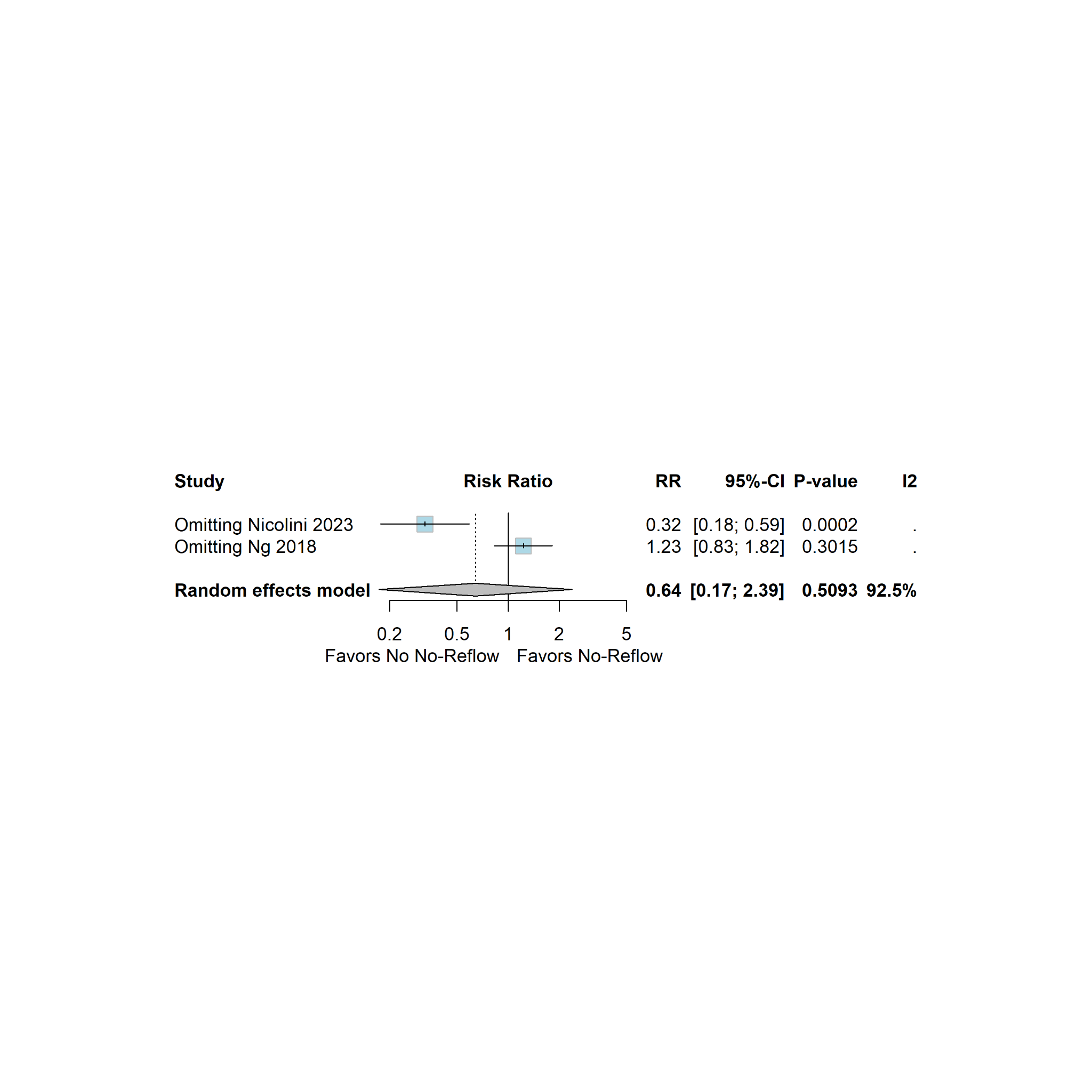


**Figure S18.** Leave-one-out sensitivity analysis for excellent functional outcome (mRS 0–1). CI: Confidence Interval; RR: Risk Ratio; I²: measure of heterogeneity.


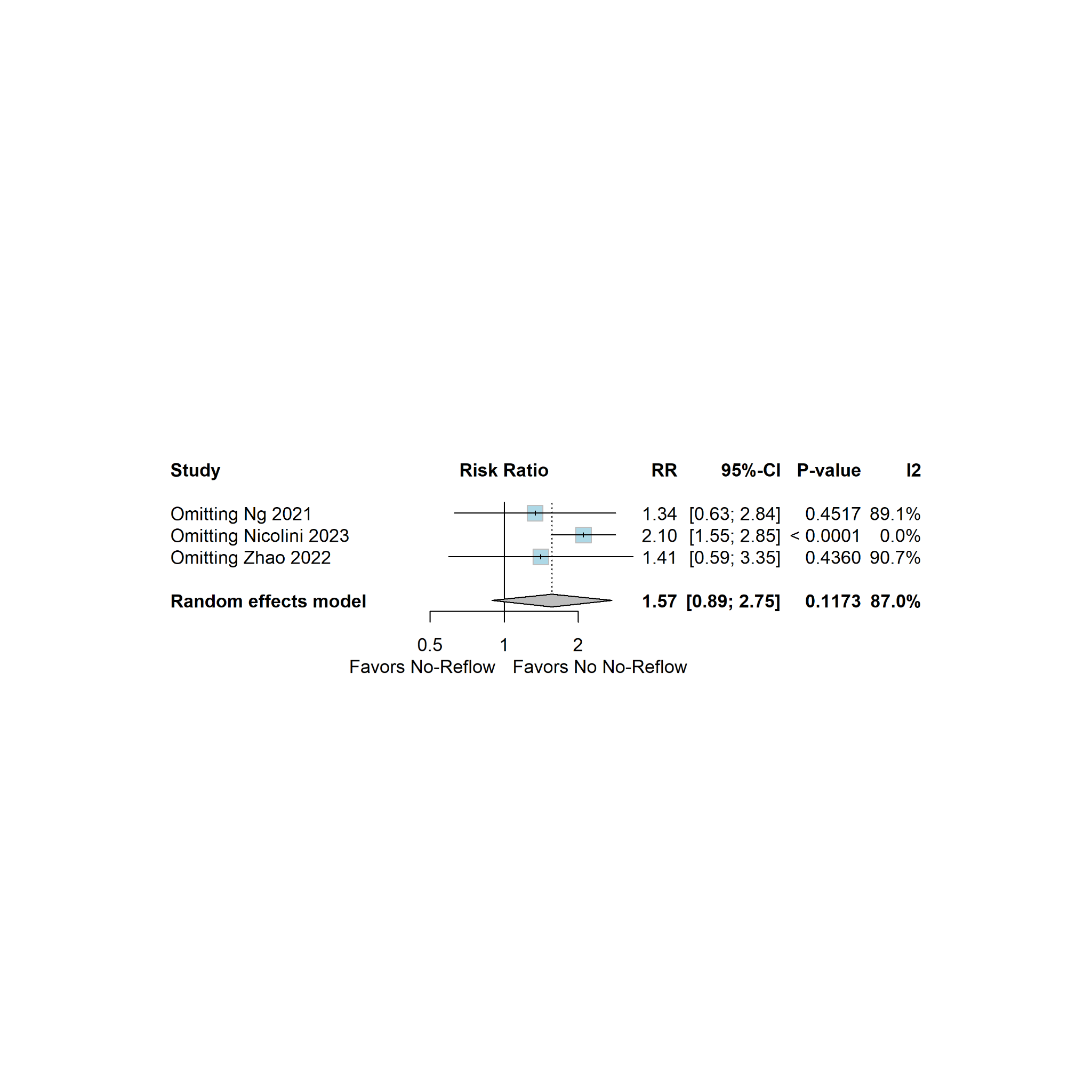


**Figure S19.** Leave-one-out sensitivity analysis for poor functional outcome (mRS 3–6). Exclusion of Nicolini et al. (2023) resulted in the lowest heterogeneity (I² = 0.0%) and a pooled RR of 2.10 (95% CI 1.55–2.85), indicating a substantial impact of this study on effect consistency.


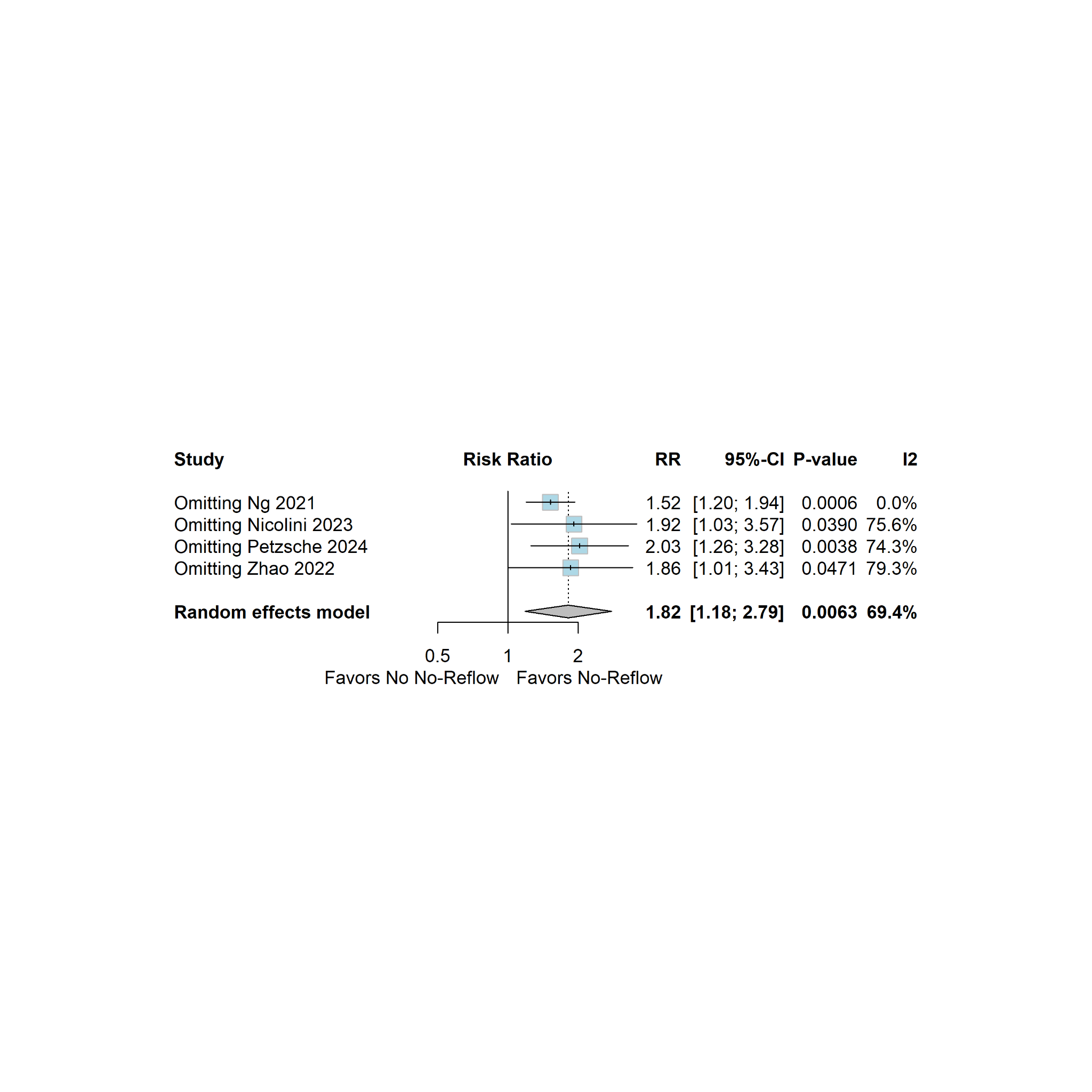


**Figure S20.** Leave-one-out sensitivity analysis for haemorrhagic transformation. Omitting Ng et al. (2021) minimized heterogeneity to 0.0%, with a corresponding RR of 1.52 (95% CI 1.20–1.94), highlighting its disproportionate influence on pooled results.

**
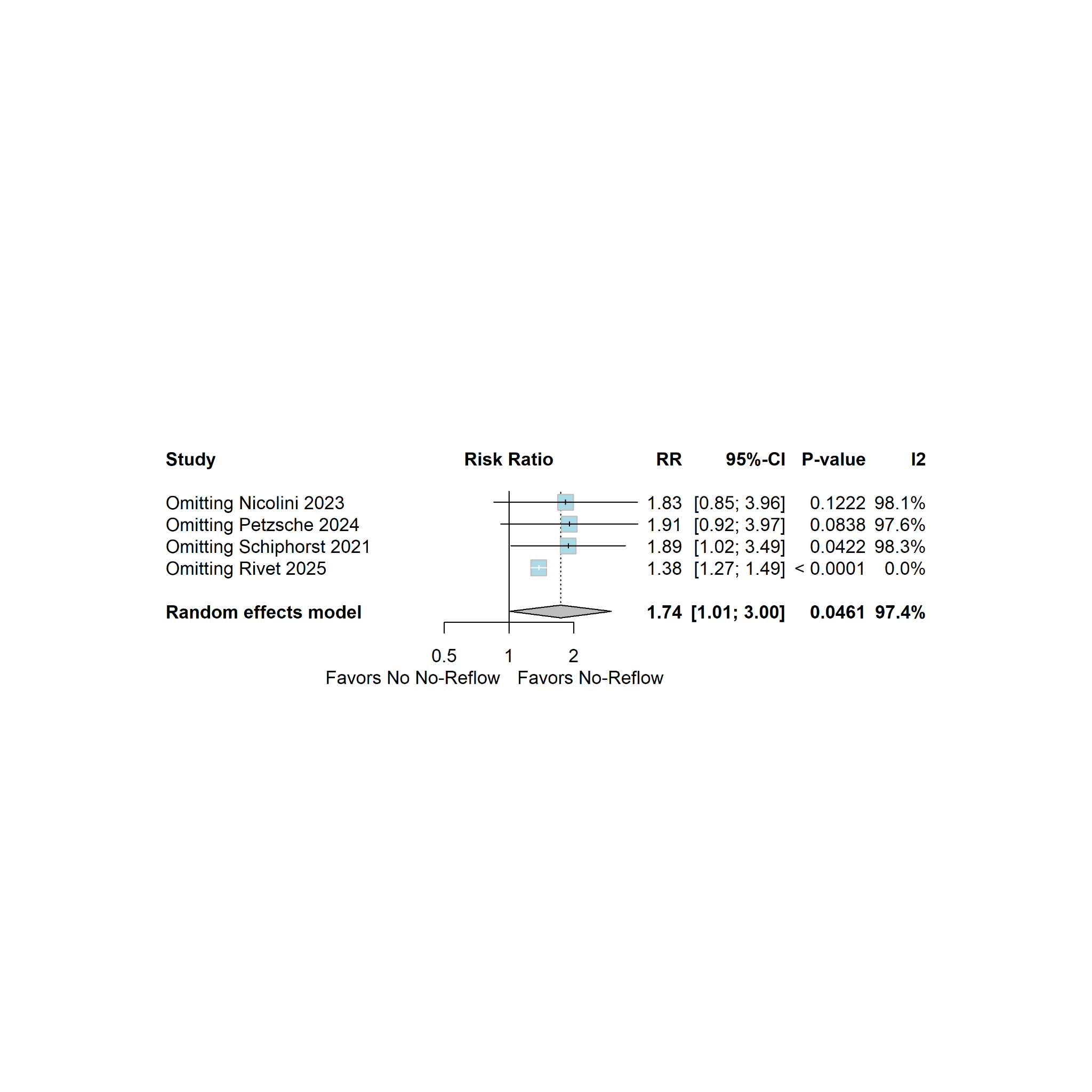
**

**Figure S21.** Leave-one-out sensitivity analysis for successful reperfusion (eTICI 2c–3). Excluding Rivet et al. (2025) led to the lowest heterogeneity (I² = 0.0%) and a refined RR of 1.38 (95% CI 1.27–1.49), indicating strong consistency across the remaining studies.

**
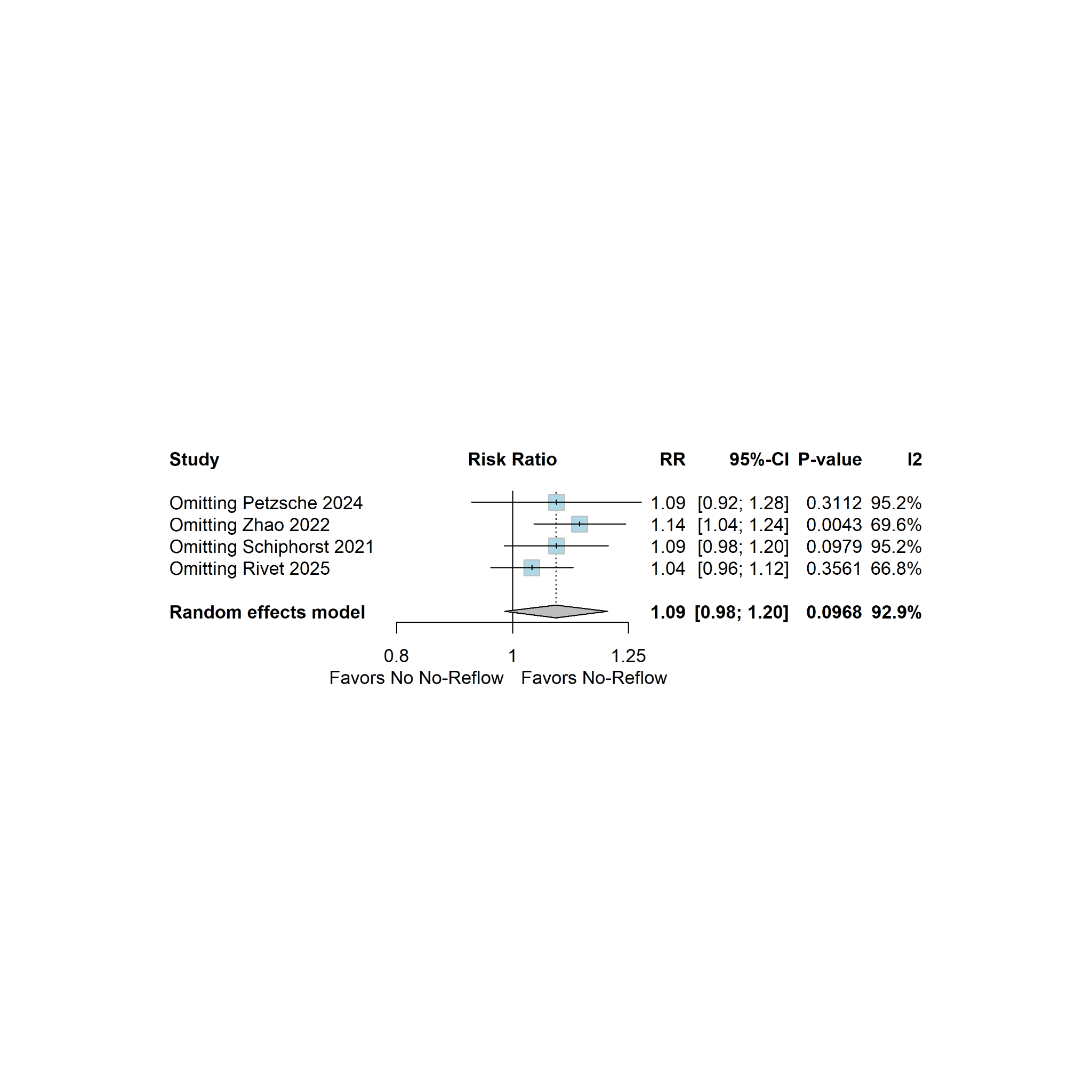
**

**Figure S22.** Leave-one-out sensitivity analysis for successful reperfusion (eTICI 2b–3). CI: Confidence Interval; RR: Risk Ratio; I²: measure of heterogeneity.

**
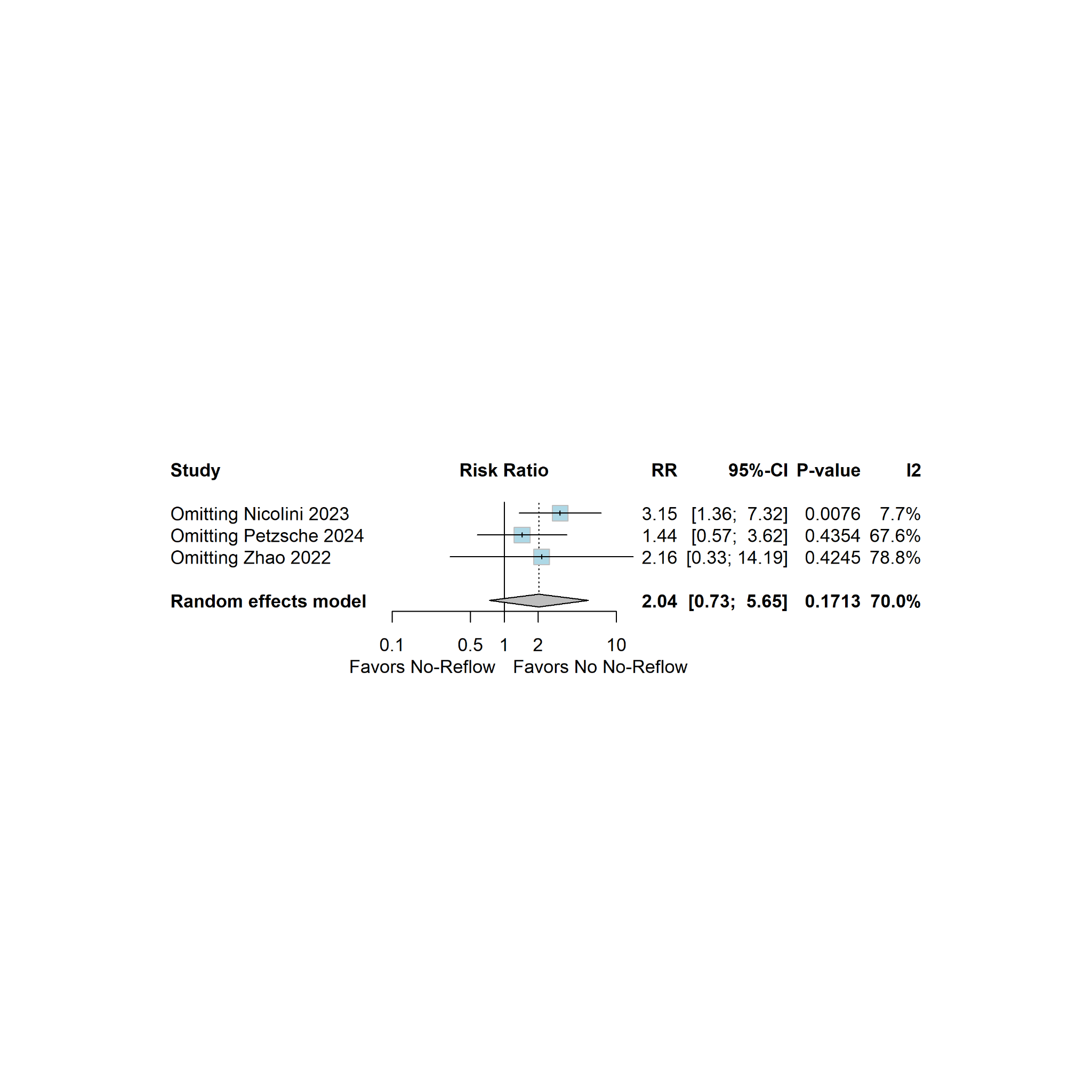
**

**Figure S23.** Leave-one-out sensitivity analysis for all-cause mortality at 90 days. Omitting Nicolini et al. (2023) yielded the lowest heterogeneity (I² = 7.7%) and an elevated RR of 3.15 (95% CI 1.36–7.32), underscoring its influential role in the pooled estimate.


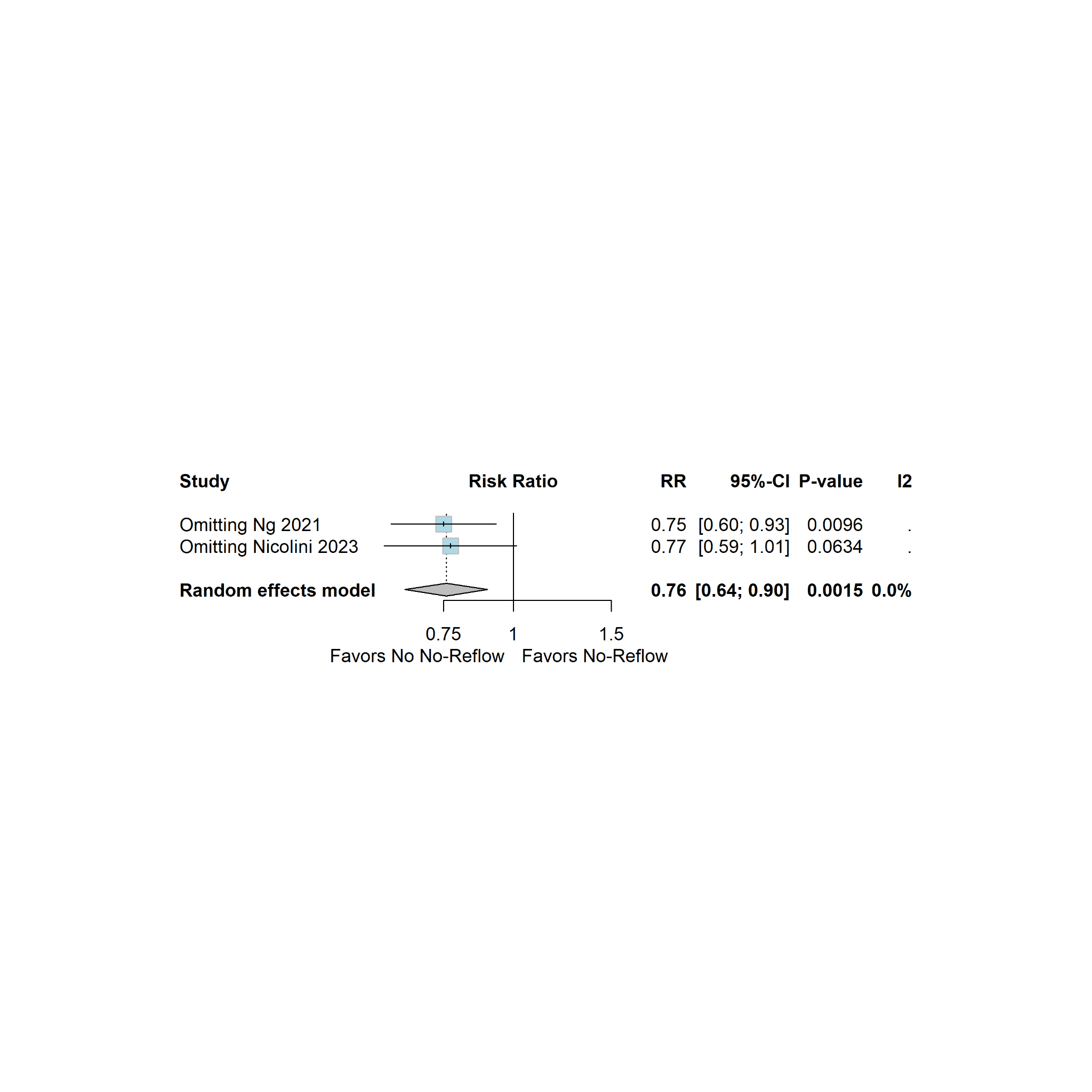


**Figure S24.** Leave-one-out sensitivity analysis for early neurologic recovery. CI: Confidence Interval; RR: Risk Ratio; I²: measure of heterogeneity.


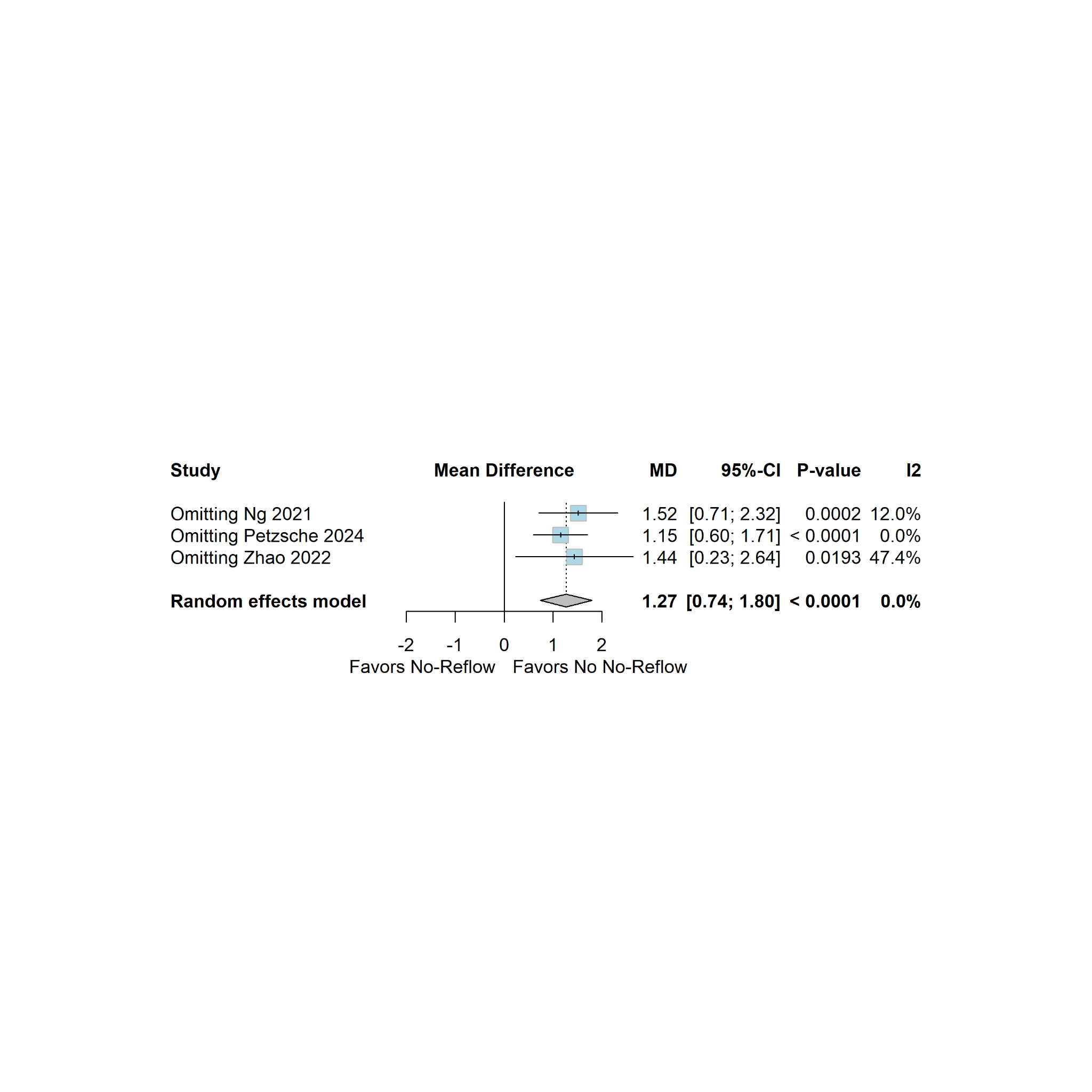


**Figure S25.** Leave-one-out sensitivity analysis for mRS variation at 90 days in patients with and without no-reflow. CI: Confidence Interval; RR: Risk Ratio; I²: measure of heterogeneity.


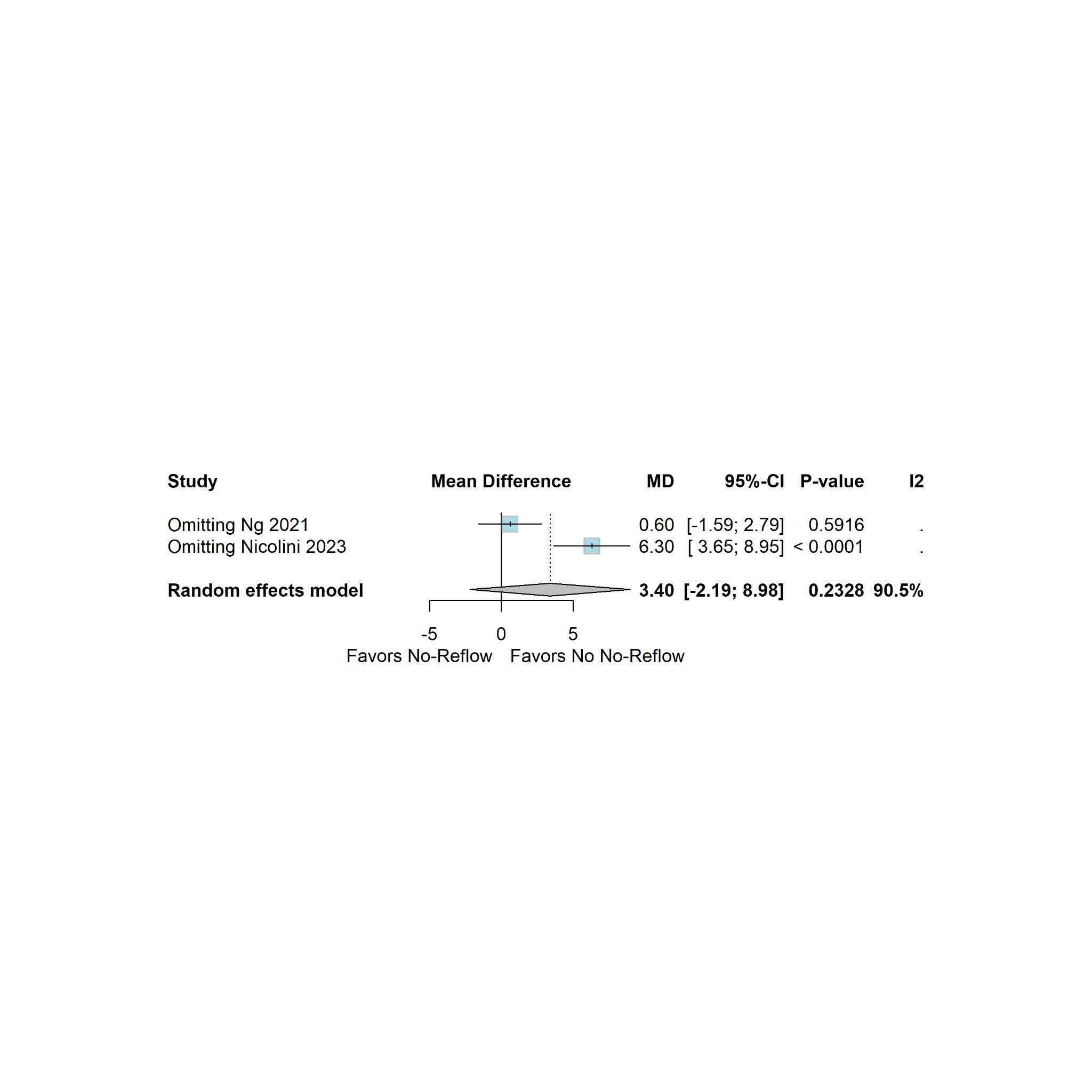


**Figure S26**. Leave-one-out sensitivity analysis for NIHSS scores at 24 hours in patients with and without no-reflow. CI: Confidence Interval; RR: Risk Ratio; I²: measure of heterogeneity.


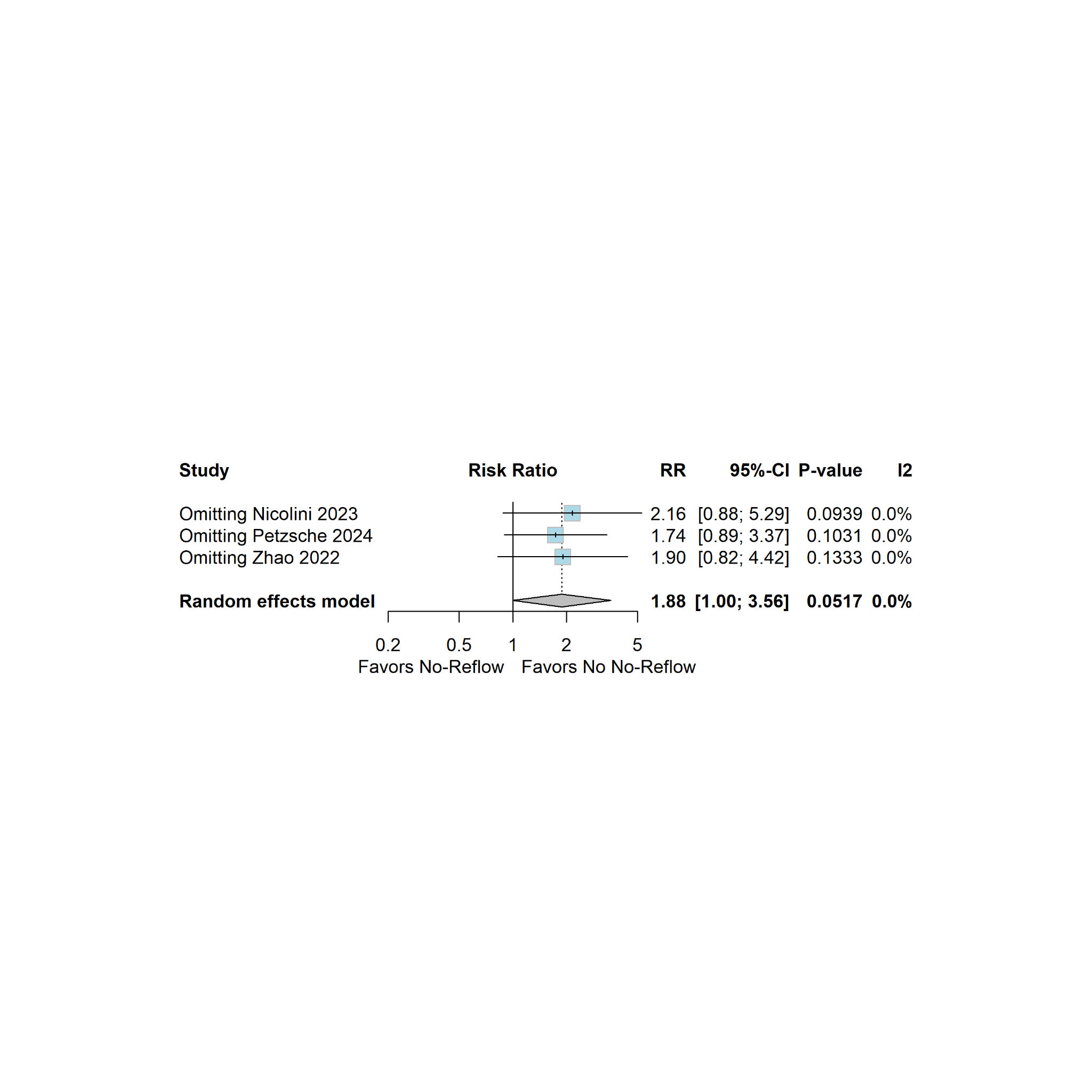


**Figure S27**. Leave-one-out sensitivity analysis for sICH in patients with and without no-reflow. CI: Confidence Interval; RR: Risk Ratio; I²: measure of heterogeneity; sICH: symptomatic Intracerebral Hemorrhage.


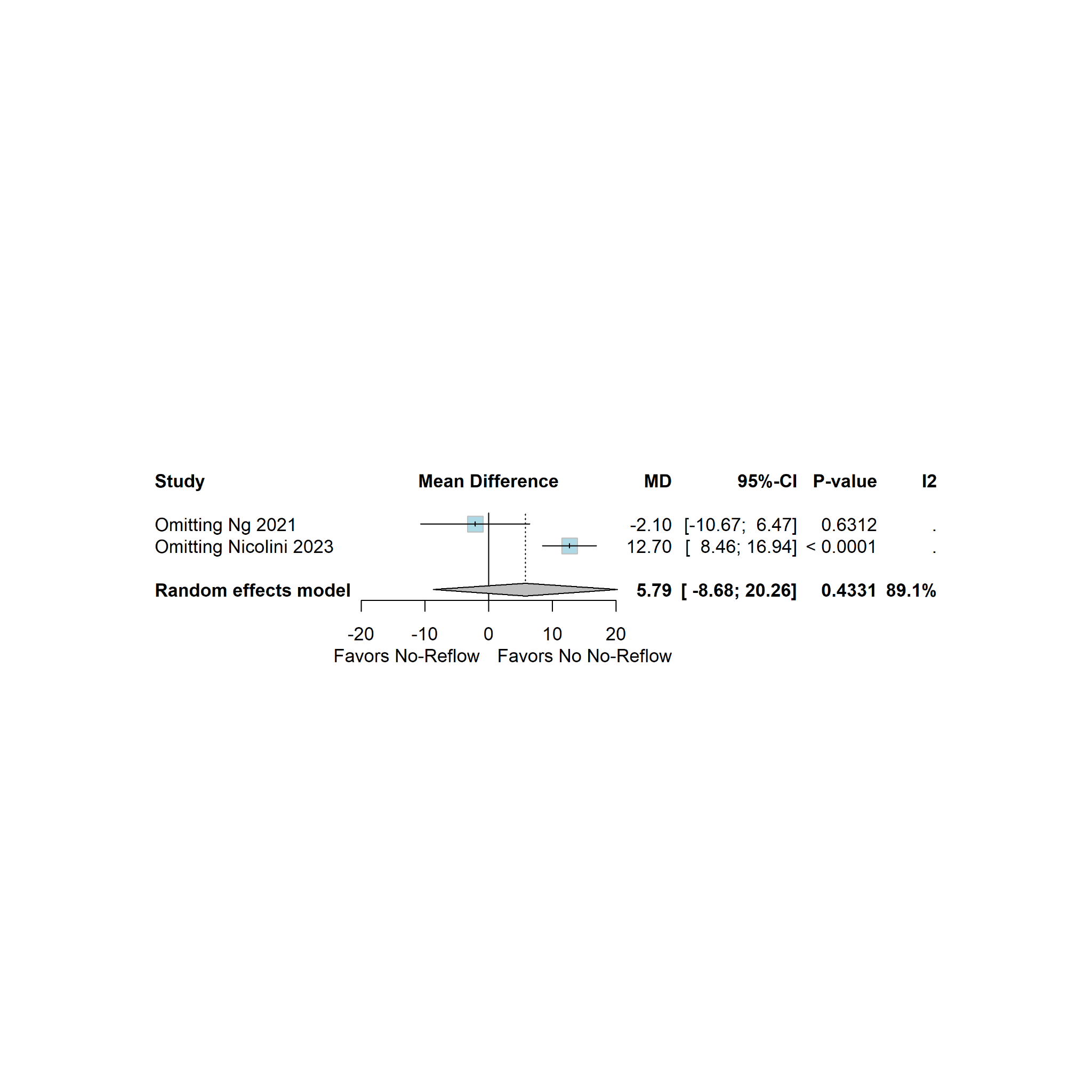


**Figure S28**. Leave-one-out sensitivity analysis for follow-up lesion volume (mL) in patients with and without no-reflow. CI: Confidence Interval; RR: Risk Ratio; I²: measure of heterogeneity; sICH: symptomatic Intracerebral Hemorrhage.

**4. Quality assessment**


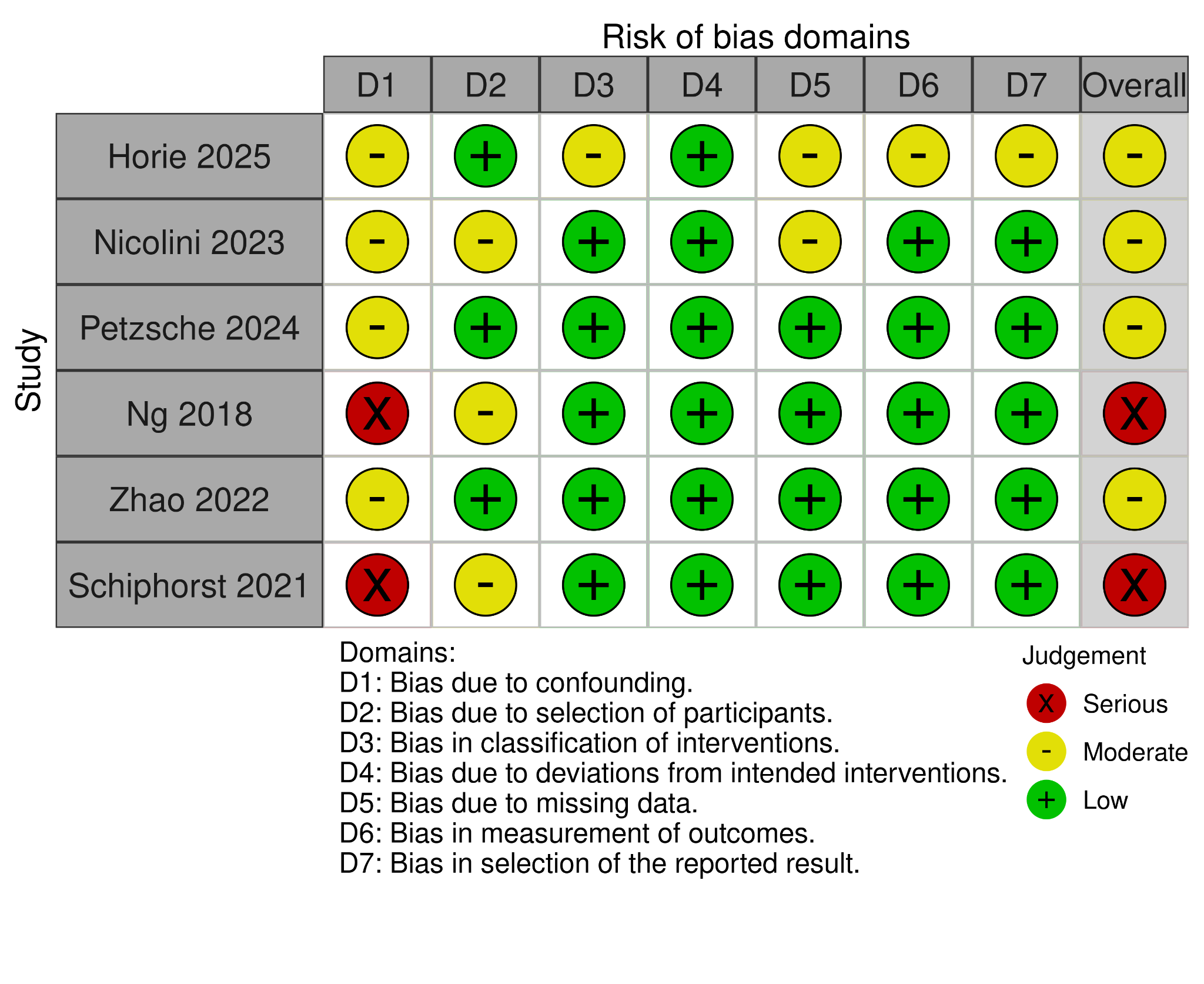


**Figure S29.** Risk of Bias Assessment (Traffic Light Plot - ROBINS-I)


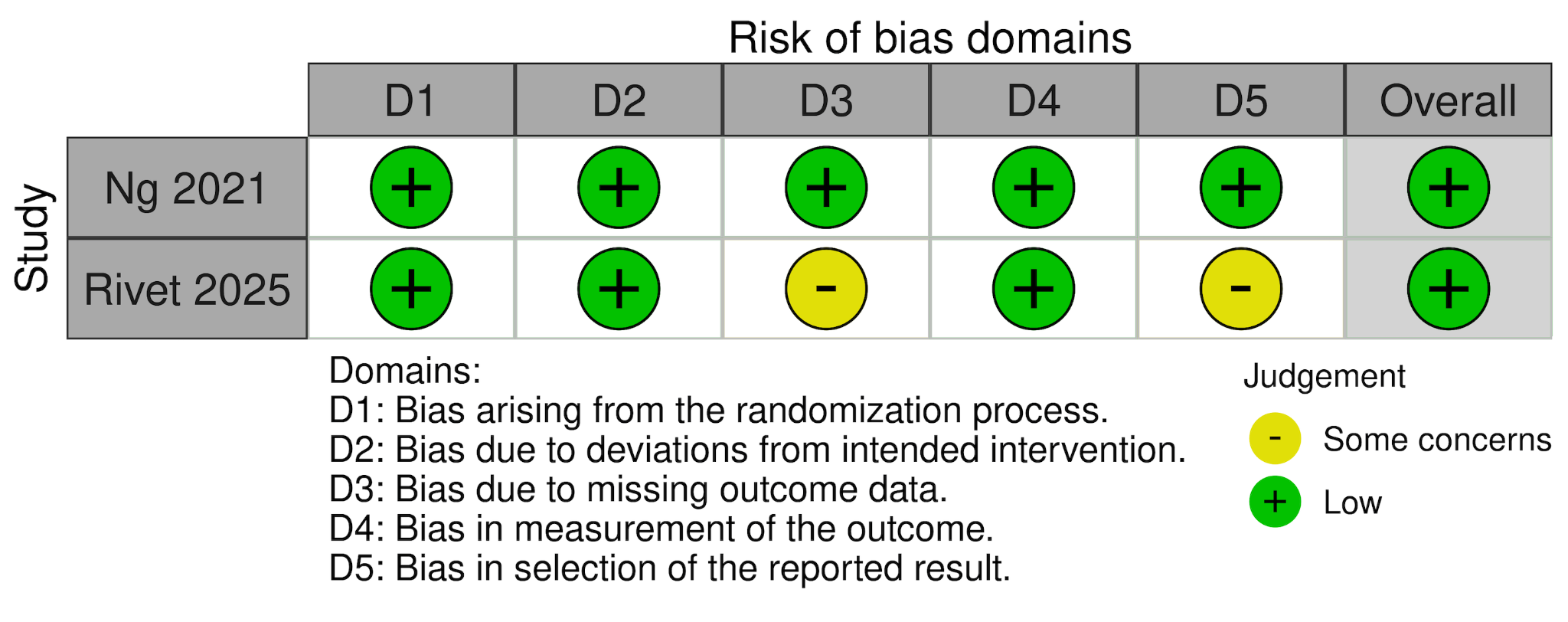


**Figure S30.** Risk of Bias Assessment (Traffic Light Plot - RoB 2.0)

**Tables**

**Table S1.** Included studies assessing no-reflow after endovascular treatment.

| **Author, y** | **No-reflow, n (%)** | **Inclusion Criteria** | **Exclusion Criteria** | **Diagnostic Modality** | **No-Reflow Definition** | **sICH Definition** |
| --- | --- | --- | --- | --- | --- | --- |
| Ng 2021 | 33 (22.4) | Anterior circulation stroke, eTICI 2c–3, perfusion imaging within 24 ± 6 h | Neurosurgery before follow-up, reocclusion at 24 h, poor image quality | Perfusion CT/MRI | Persistent hypoperfusion on perfusion imaging at 24 h | NA |
| Horie 2025 | 52 (80.0) | ICA or MCA (M1/M2) occlusion, successful thrombectomy (TICI ≥2b) | Chronic occlusion, dissection, contralateral lesions | Perfusion imaging (Tmax map) | Tmax >6 s on perfusion imaging | NA |
| Nicolini 2023 | 65 (35.1) | LVO in anterior circulation, mTICI 2c–3, DSA post-intervention available | ACA occlusion, incomplete angiographic phases, missing data | DSA | mCIS >3 modified capillary index score | Type 2 parenchymal hemorrhage associated with an increase of ≥ 4 points on the NIHSS at 24 h or leading to death |
| Petzsche 2024 | 10 (9.0) | Age ≥18 years, anterior LVO stroke, MT performed, MRI within 10 days | Posterior stroke, poor imaging, MRI contraindications | MRI perfusion | Hypoperfusion >50% infarct core, CBF reduction >15% | Secondary neurological deterioration of ≥ 4 points on the NIHSS scale and evidence of cerebral hemorrhage on imaging |
| Ng 2018 | 53 (50.0) | MCA M1/proximal M2 occlusion, EVT within 6 h, TICI 2b–3, TCD ≤3 days | Posterior or ACA occlusion, late TCD, TCD contraindications | TCD | MCA-PI >1.2 | NA |
| Zhao 2022 | 45 (26.5) | MCA M1 occlusion, EVT, TICI 3, TCD within 24 h | ICA/MCA stenosis, no TCD, poor windows, reocclusion | TCD | MCA-PI >1.09 (highest quartile) | Any apparently extravascular blood in the brain or within the cranium that was associated with clinical deterioration, as defined by an increase of 4 points or more in the score on the NIHSS, or that led to death, was identified as the predominant cause of the neurological deterioration |
| Schiphorst 2021 | 1 (3.0) | ICA or M1 occlusion, complete recanalization (mTICI 2c–3), ASL MRI at 24 h | Parenchymal hematoma, reocclusion, ICA stenosis ≥50%, MRI delay >3 h | ASL MRI | Severe hypoperfusion on ASL plus infarction | NA |
| Rivet 2025 | 30 (20.0) | LVO stroke, eTICI 2c–3, perfusion CT/MRI at 24 h | Posterior strokes, no perfusion, reocclusion, ICA stenosis >50% | Perfusion CT/MRI | Hypoperfusion >15% vs contralateral ROI on perfusion scan | NA |

*Abbreviation: ASL: arterial spin labeling; CBF: cerebral blood flow; CT: computed tomography; DSA: digital subtraction angiography; EVT: endovascular treatment; ICA: internal carotid artery; LVO: large vessel occlusion; MCA: middle cerebral artery; MRI: magnetic resonance imaging; MT: mechanical thrombectomy; PI: pulsatility index; ROI: region of interest; TCD: transcranial Doppler; Tmax: time to maximum of the residue function; TICI: thrombolysis in cerebral infarction scale; mRS: modified. Rankin Scale.*

**6. Funnel Plot analysis**


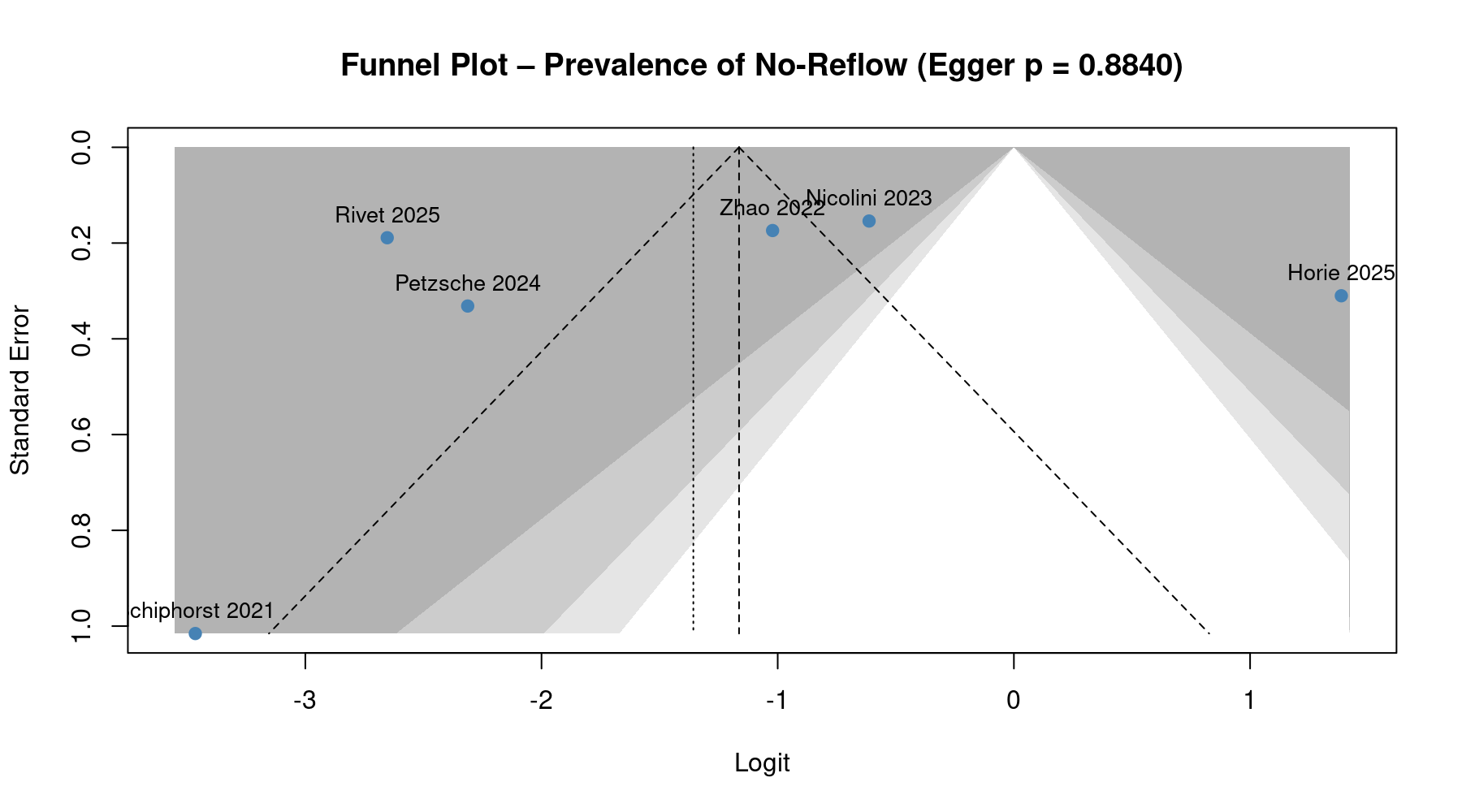


**Figure S31.** Funnel Plot analysis of the Prevalence of No-Reflow in the studies.


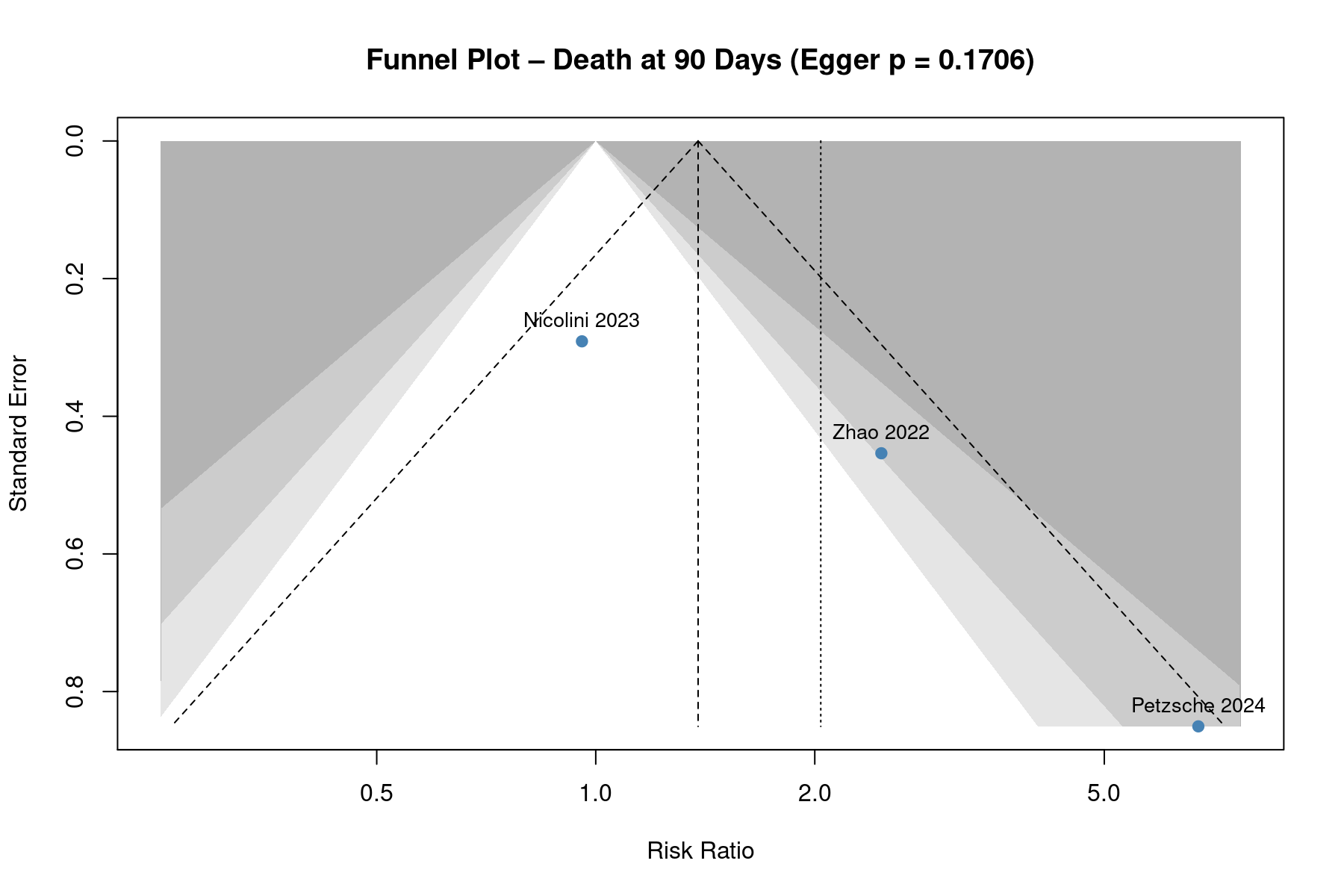


**Figure S32.** Funnel Plot analysis of Death at 90 days.


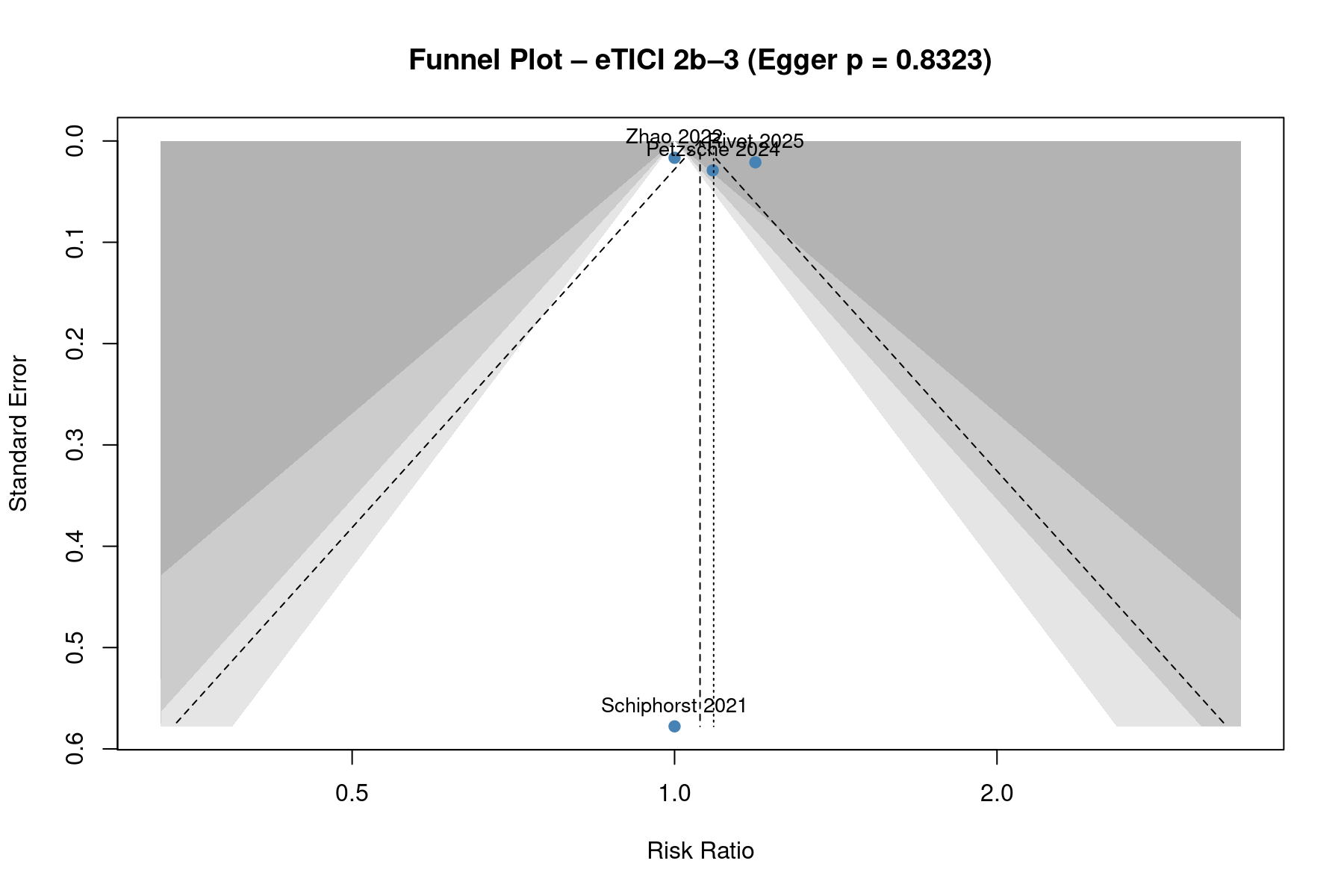


**Figure S33.** Funnel Plot analysis of eTICI 2b-3 (eTICI: Expanded Thrombolysis in Cerebral Infarction).


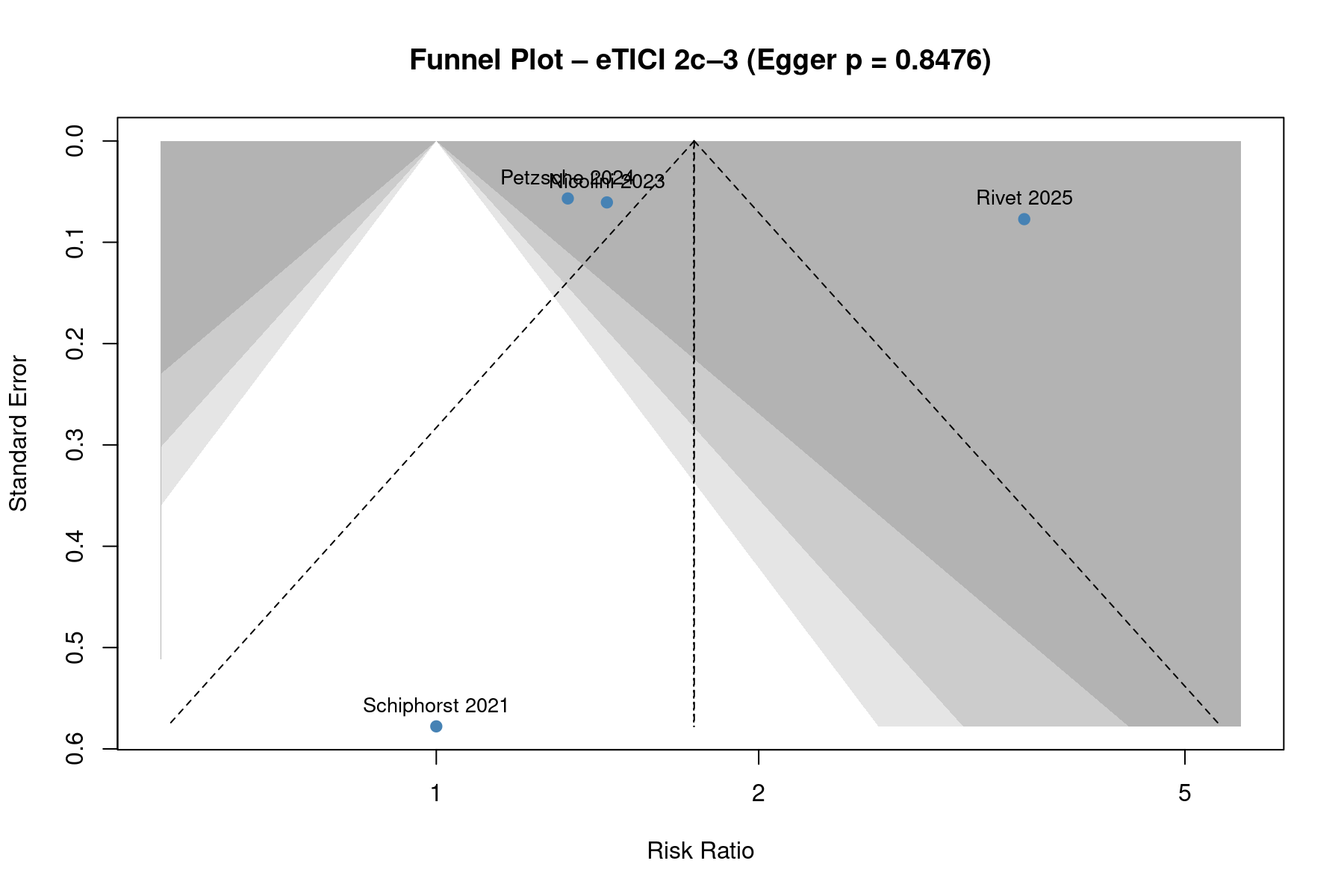


**Figure S34.** Funnel Plot analysis of eTICI 2c-3 (eTICI: Expanded Thrombolysis in Cerebral Infarction).


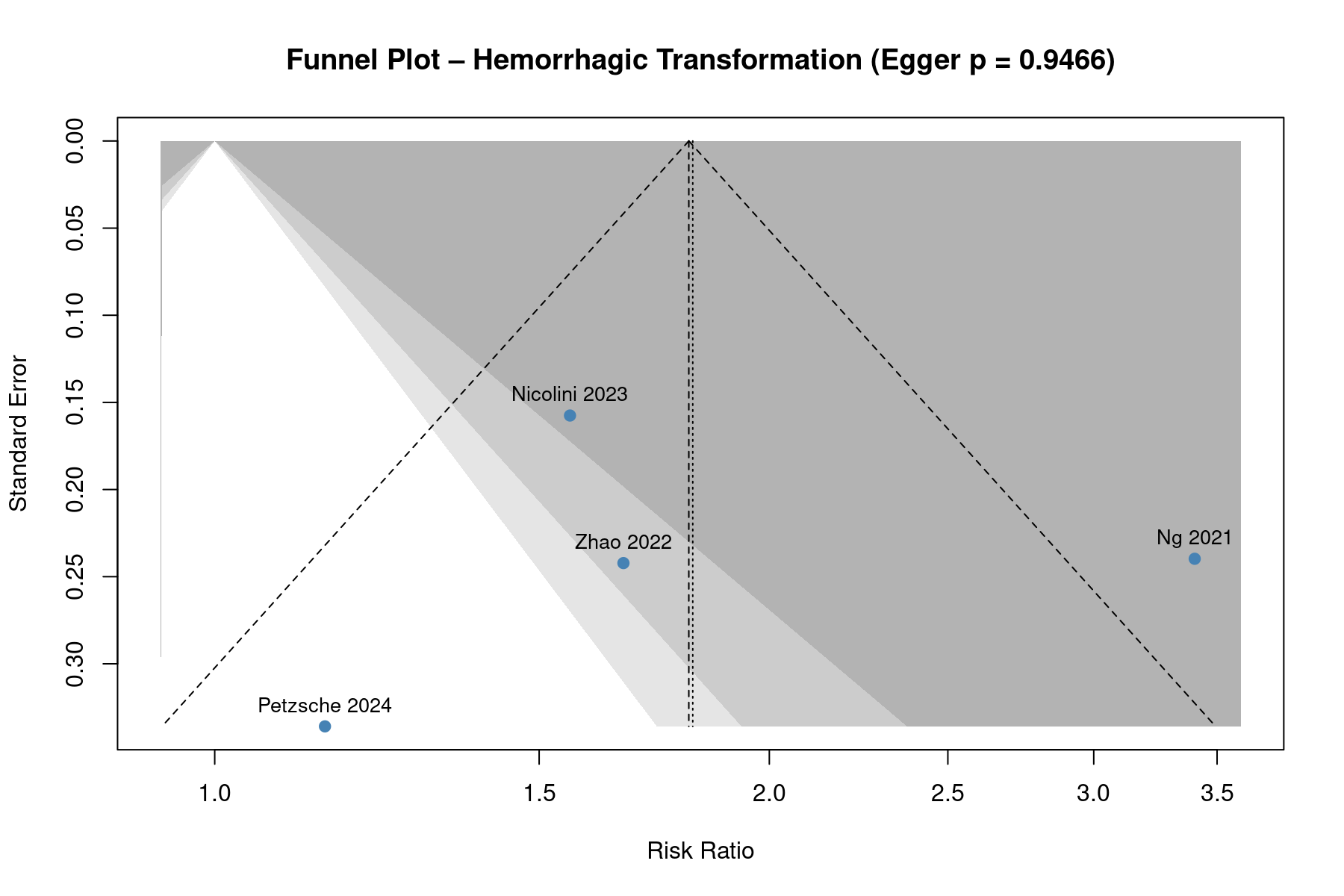


**Figure S35.** Funnel Plot analysis of Haemorrhagic Transformation.


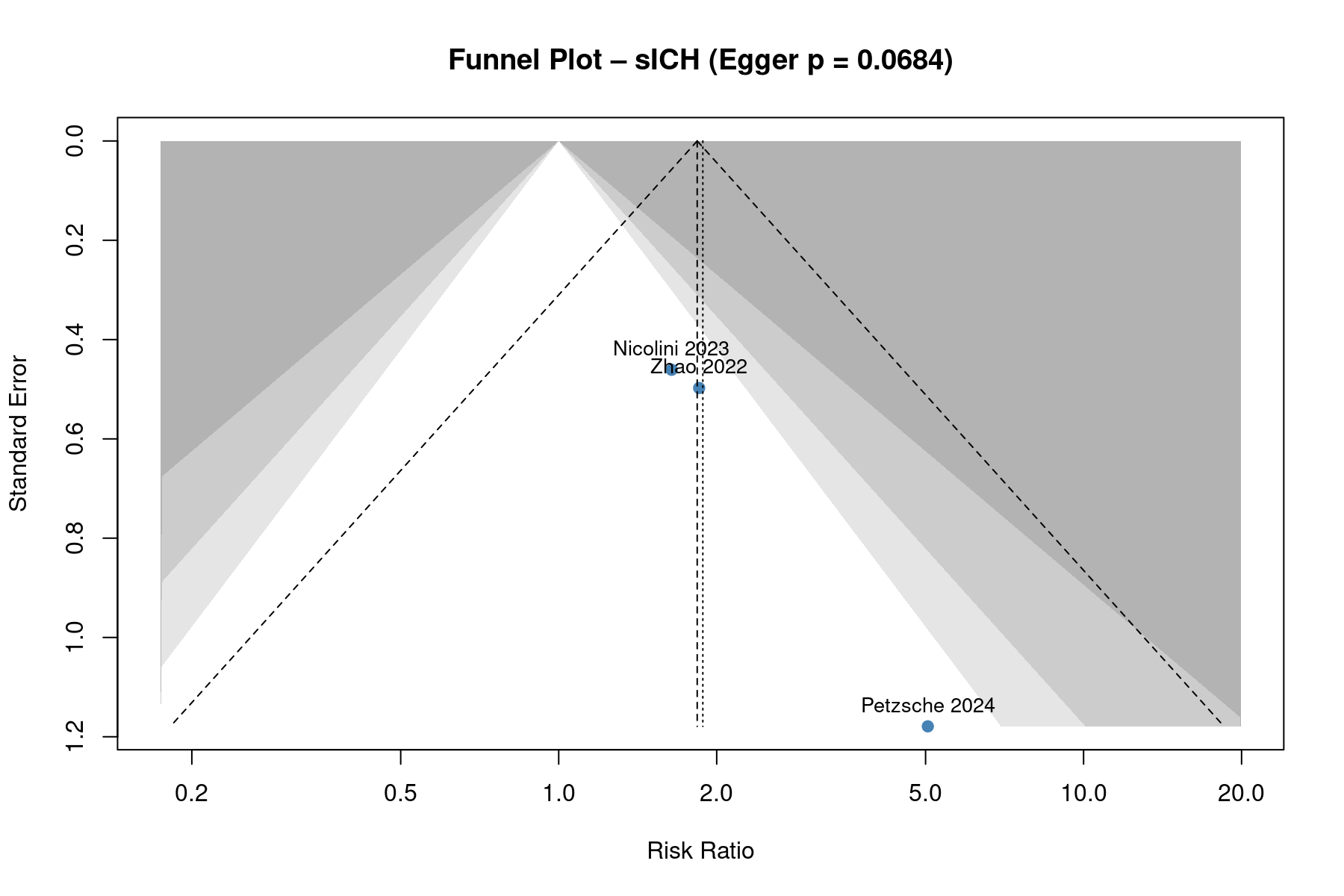


**Figure S36.** Funnel Plot analysis of sICH (symptomatic intracerebral hemorrhage).
